# Assessing the ability of sequence-based methods to provide functional insight within membrane integral proteins: a case study analyzing the neurotransmitter/Na^+ ^symporter family

**DOI:** 10.1186/1471-2105-8-397

**Published:** 2007-10-17

**Authors:** Dennis R Livesay, Patrick D Kidd, Sepehr Eskandari, Usman Roshan

**Affiliations:** 1 Department of Computer Science and Bioinformatics Research Center, University of North Carolina at Charlotte, Charlotte, NC 28262, USA; 2 Biological Sciences Department, California State Polytechnic University, Pomona, CA 91768, USA; 3 Department of Computer Science, New Jersey Institute of Technology, Newark, NJ 07102, USA

## Abstract

**Background:**

Efforts to predict functional sites from globular proteins is increasingly common; however, the most successful of these methods generally require structural insight. Unfortunately, despite several recent technological advances, structural coverage of membrane integral proteins continues to be sparse. ConSequently, *sequence-based *methods represent an important alternative to illuminate functional roles. In this report, we critically examine the ability of several computational methods to provide functional insight within two specific areas. First, can phylogenomic methods accurately describe the functional diversity across a membrane integral protein family? And second, can sequence-based strategies accurately predict key functional sites? Due to the presence of a recently solved structure and a vast amount of experimental mutagenesis data, the neurotransmitter/Na^+ ^symporter (NSS) family is an ideal model system to assess the quality of our predictions.

**Results:**

The raw NSS sequence dataset contains 181 sequences, which have been aligned by various methods. The resultant phylogenetic trees always contain six major subfamilies are consistent with the functional diversity across the family. Moreover, in well-represented subfamilies, phylogenetic clustering recapitulates several nuanced functional distinctions. Functional sites are predicted using six different methods (phylogenetic motifs, two methods that identify subfamily-specific positions, and three different conservation scores). A canonical set of 34 functional sites identified by Yamashita et al. within the recently solved LeuT_Aa _structure is used to assess the quality of the predictions, most of which are predicted by the bioinformatic methods. Remarkably, the importance of these sites is largely confirmed by experimental mutagenesis. Furthermore, the collective set of functional site predictions qualitatively clusters along the proposed transport pathway, further demonstrating their utility. Interestingly, the various prediction schemes provide results that are predominantly orthogonal to each other. However, when the methods do provide overlapping results, specificity is shown to increase dramatically (e.g., sites predicted by any three methods have both accuracy and coverage greater than 50%).

**Conclusion:**

The results presented herein clearly establish the viability of sequence-based bioinformatic strategies to provide functional insight within the NSS family. As such, we expect similar bioinformatic investigations will streamline functional investigations within membrane integral families in the absence of structure.

## Background

Due to their immense biomedical importance, as well as their strong representation within genomes [1], membrane channels and transporters are among the most important classes of proteins to better understand. These proteins facilitate movement of substrates (i.e., metalloions, amino acids, signaling molecules, etc.) across intervening membrane barriers. Historically, our understanding of these proteins has been hampered by a lack of structural information. However, starting with the potassium channel, which is a membrane transport protein whose structure was solved at high resolution [2], there has been a string of recently solved structures of transporter proteins, including the ATP binding cassette (ABC) transporter [3], the multidrug efflux transporter AcrB [4], lac-permease [5], aquaporin [6], the glutamate transporter [7], ammonia channel AmtB [8], Na^+^/H^+ ^antiporter [9], and recently, a leucine transporter, LeuT_Aa _[10], which is a bacterial member of the Na^+^- and Cl^-^-coupled family of transporters (SLC6 according to the Human Genome Organization classification). From these groundbreaking efforts, our overall understanding of the sequence/structure/function relationships within transporter proteins is beginning to mature to a point where accurate descriptions of mechanism are possible [11-15]. Unfortunately, despite these successes, structural coverage of this segment of the proteome will continue to be sparse for the foreseeable future.

LeuT_Aa _is a good example where a solved structure has provided a framework to investigate function within its biomedically important homologs [10]. LeuT_Aa _is a bacterial member of the Na^+^/Cl^- ^dependent transporter family, which is also called the neurotransmitter/Na^+ ^symporter family (NSS; 2.A.22 according to the transporter classification system) [16-20]. In the NSS family, free energy provided by the flux of sodium and chloride ions down their electrochemical gradients is used to move chemical substrates against concentration gradients across a membrane barrier [21-24]. The chemical substrates recognized by members of the NSS family are extremely diverse and include amino acids, biogenic amines, and osmolytes. For example, the serotonin transporter, which is localized to the presynaptic terminal plasma membrane and is responsible for recycling serotonin to the releasing neuron, is a member of this functionally diverse family [25]. The serotonin transporter is responsible for clearing serotonin from the synapse after neurotransmitter release, and is the target of many current anti-depression drugs [26]. Other members of the family include transporters of dopamine, norepinephrine, γ-aminobutyric acid (GABA), glycine, proline, creatine, betaine, taurine, and several other small-molecule substrates [17]. Similar to the serotonin transporter, there is substantial clinical interest in the dopamine, norepinephrine, GABA, and glycine transporters. Although the family includes many ORFans (sequences that have not been functionally annotated), subfamily differentiation is generally consistent with the chemical diversity of the transported molecules [10,17].

The LeuT_Aa _protein structure is composed of twelve transmembrane helices, two intracellular helices, four extracellular helices, and a small extracellular β-hairpin [10]. At present, the fold has only been observed within the NSS family. A lack of an unobstructed path through the structure indicates that the structure was solved in the "closed" or "occluded" state [27]. Interestingly, the first and sixth transmembrane helices (TM1 and TM6) are partially unwound. It has been hypothesized that these unwound regions provide hinges that allow the structure to cycle between three distinct conformational states: outward facing open ↔ closed ↔ inward facing open [10]. The backbone amides and carbonyls of the extended residues are involved in ion coordination and hydrogen bonding to the leucine substrate.

Due to the paucity of structural coverage, bioinformatic methods provide an attractive means to guide functional studies of membrane integral proteins. In this report, we assess the ability of several *sequence-based *bioinformatic tools to predict key functional sites within the NSS family. The LeuT_Aa _structure provides structural hindsight to gauge the accuracy of the predictions. Our results clearly establish that sequence-based methods can provide key residue-level insight into the structurally derived set of functional sites. Remarkably, the importance of these sites is corroborated by previous mutagenesis experiments done on the serotonin, dopamine, and GABA transporters. In addition, this report provides a more complete picture of functional divergence within the NSS family than previously described. Using a phylogenomics approach, several ORFan NSS members have been annotated and subfamily differentiation is shown to parallel known functional distinctions.

## Results and Discussion

### Familial phylogeny and phylogenomic assignment of function

**Figure 1:**
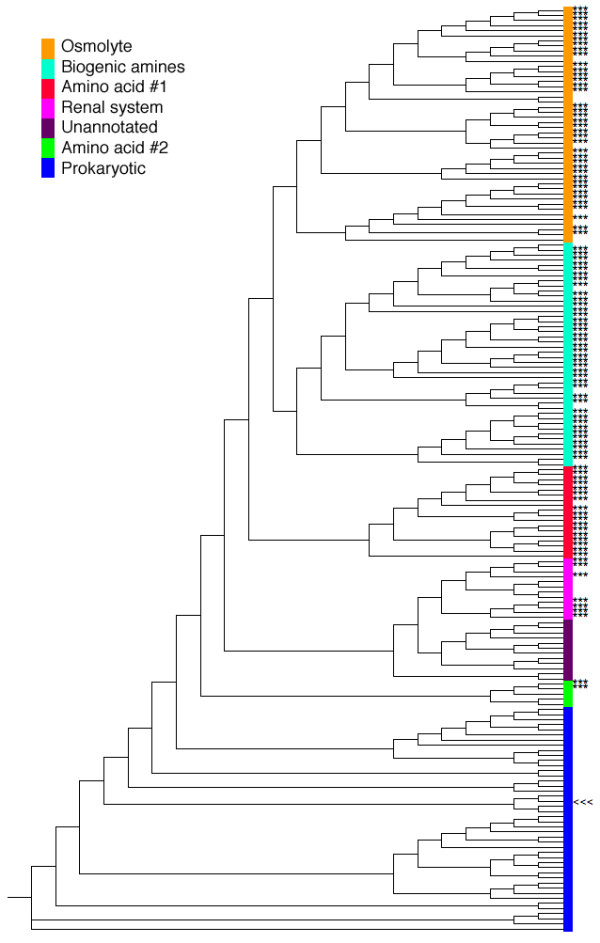
Phylogenetic tree of the complete NSS family. The tree is composed of six major distinct subfamilies, four of which are specifically associated with a specific chemical class of substrates (osmolytes, biogenic amines, and two distinct classes of amino acids). Only 7 of the 24 sequences within the subfamily generically annotated as Renal system (light purple) have been experimentally characterized. The dark purple subfamily lacks any experimental annotation; however, we include it within the Renal system subfamily based on the location of the branch point. The sixth subfamily (generically annotated as prokaryotic) is much more divergent. In fact, it appears that the prokaryotic subfamily could be further split, indicated by the light and dark shades of blue. However, we do not do so due to the lack of functional annotation discriminating between the two. Triple asterisks indicate leaves of experimentally annotated homologs; the other highlighted leaf (<<<) corresponds to the sequence of the LeuT_Aa _structure.

The ClustalW [28] generated NSS family phylogenetic tree is shown in Figure 1 and provided in Additional file 1. The tree has six major subfamilies (see Table 1). The PHYLIP [29] generated tree (not shown) has only minor topological differences; all six subfamily bipartitions are conserved within each tree. Four of the six subfamilies are associated with substrates of specific chemical classes. These four subfamilies include transporters for: biogenic amines (dopamine, norepinephrine and epinephrine, and serotonin), osmolytes (GABA, betaine, taurine, creatine, and several ORFans), as well as two evolutionarily distinct classes of amino acid transporters (designated Amino acid #1 and Amino acid #2). The other two subfamilies include a poorly characterized subfamily generically designated as the Renal system (because most of the characterized sequences from this subfamily are found within the kidney and/or intestine), and a large prokaryotic subfamily. The osmolyte subfamily, which is the largest subfamily observed, contains 46 sequences. Conversely, the second of two amino acid subfamilies (Amino acid #2) has only five sequences. Bootstrapping clearly indicates that all six subfamilies (in both trees) are statistically robust, including the small Amino acid #2 subfamily (see Additional file 2).

**Table 1:** Summary of subfamilies identified within the NSS family.^1^

**Subfamily Designation**	**Description**	**Number of Sequences**
Osmolytes	Two distinct groups of GABA transporters (11 and 12, respectively). Also includes: creatine (6), taurine (9), and betaine transporters (6). Two remain ORFans.	46
Biogenic amines	Two distinct groups of dopamine transporters (13 and 6, respectively). Also includes: norepinephrine (13), epinephrine (1), and serotonin (11) transporters.	44
Renal system	Only 7 sequences are characterized, of which: 5 are isolated from the renal system; 1 is an intestinal brush border proline transporter; and the last was found in the brain. Due to the location of the branch point, the sequences highlighted in dark purple in Figure 1 are also included; however, no experimental evidence is available to confirm or refute this.	24
Amino acid #1	Mainly includes proline (5) and glycine (10) transporters; 3 are generically annotated as "amino acid" transporters.	18
Amino acid #2	3 generically annotated as "amino acid" transporters and 2 ORFans.	5
Prokaryotic	Bacterial and archaeal transporters (includes LeuT_Aa_). Nearly all are from genome sequencing projects and lack robust biophysical characterization of functional specificity.	44

^1 ^An exact summary of all experimentally characterized and ORFan sequences are provided in Additional file 1.

**Figure 2:**
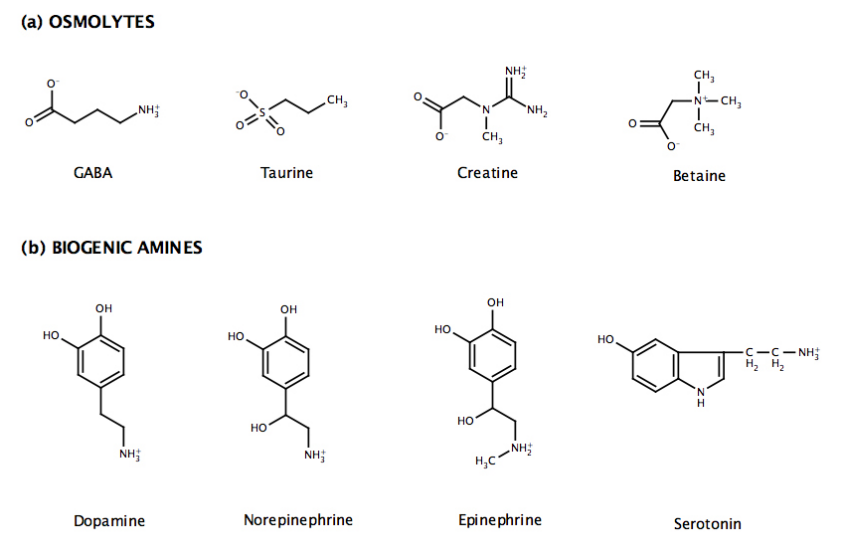
Chemical diversity of the osmolytes and biogenic amines. **(a) **All of the osmolytes are of similar size and are zwitterionic. Moreover, the separation of charge in each is nearly equal. **(b) **The biogenic amines (which are common neurotransmitters within the brain) are all aromatic amines. As one would expect based on the chemical diversity within the biogenic amines, there is an evolutionary split between the serotonin and catecholamine transporters.

**Figure 3:**
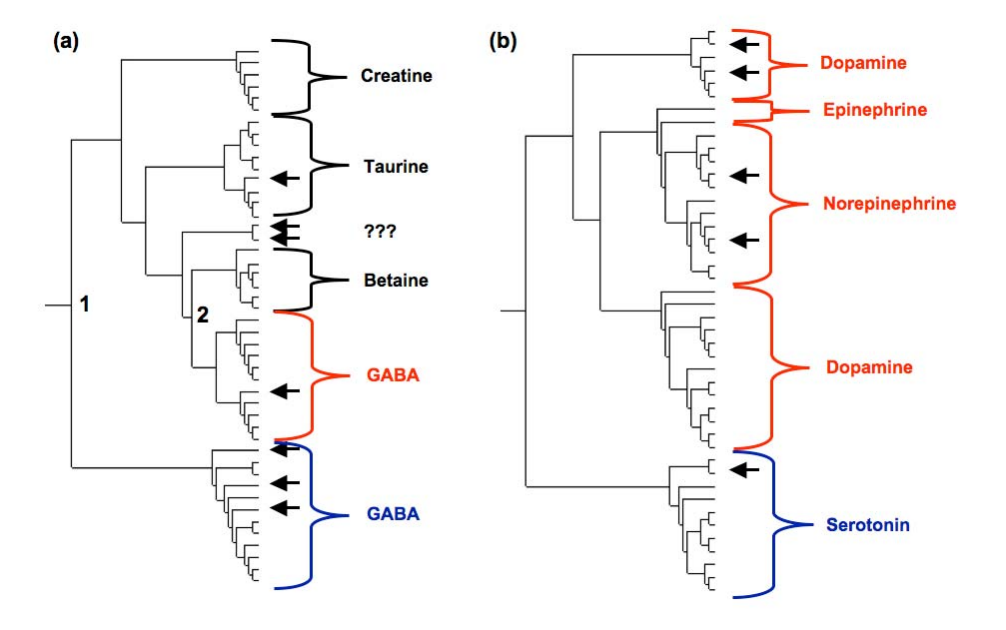
Expansion of the **(a) **osmolyte and **(b) **biogenic amine portions of the phylogenetic tree. Seven of the 46 osmolyte sequences remain experimentally uncharacterized (indicated by arrows). Annotations of the remaining sequences are indicated. Using a phylogenomic approach, we assign functions to five of the seven. The remaining pair (indicated by question marks) is still without functional annotation. Similarly, there are five uncharacterized sequences within the biogenic amine subfamily; we have assigned function to all five. Branch points 1 and 2 designate the differentiation of the two GABA symporter out-groups. Color differences within each out-group correspond to functional distinctions discussed within the text.

While there are significant chemical differences among the substrates across the entire NSS family, differences within subfamilies are greatly diminished. For example, all of the four known substrates within the osmolyte subfamily are of similar size and are zwitterionic (Figure 2a). Moreover, the spatial separation of charge within each is also fairly conserved. In order to extend the annotations beyond the known (experimental) descriptions, we employ a phylogenomics approach [30,31], where appropriate, to assign functional specificity to sequences without annotation. ORFans within otherwise obviously annotated out-groups are associated with the consensus annotation. For example, in the osmolyte subfamily, seven sequences are annotated as ORFans (arrows in Figure 3b). Five of those sequences occur within well-established out-groups. ConSequently, functional annotations are assigned here based on the other sequences within their respective out-group. The remaining two ORFans occur together, but do not fall into any obvious substrate distinction. As such, these two sequences remain unannotated (question marks in Figure 3a). Application of this approach to the entire NSS family increases the number of functional annotations by 12, which is significant, but not necessarily remarkable. However, the annotation improvement becomes significantly more impressive when investigating only the osmolyte and biogenic amine transporter subfamilies, both of which are better characterized experimentally [20,23,26,32-35]. In these two examples alone, ten of twelve ORFans can be functionally classified using phylogenetics. (Note: a complete list of all experimentally characterized sequences, ORFans, and the twelve newly annotated sequences is provided in Additional file 2.)

Curiously, there are two distinct groups of GABA transporters within the osmolyte subfamily. The first GABA out-group diverged from the rest of the osmolyte subfamily (designated as branch point 1 in Figure 3a). Branch point 2 diverged into the second GABA out-group and the betaine out-group. It is known that some GABA symporters can transport both GABA and betaine [32,36]. Therefore, it is tantalizing to suggest based solely on the phylogeny that it is the second group of GABA symporters (shown in red in Figure 3a) that can transport both GABA and betaine, whereas the first group is specific to GABA only (shown in blue in Figure 3a). This is, in fact, supported by experimental data. Members of this second GABA transporter out-group have been shown to exhibit substrate overlap with members of the other branches in the tree. For example, mouse GAT2 (homolog of BGT-1) transports betaine, and mouse GAT3 (homolog of rat/human GAT-2) and mouse GAT4 (homolog of rat/human GAT-3) transport taurine and creatine (Spencer, Padilla, and Eskandari, unpublished). GAT1 (first GABA transporter out-group) does not transport betaine, creatine, or taurine [32]. This result profoundly demonstrates the ability of phylogenomic methods to provide key insight regarding the rise of functional specificity divergence.

Due to its association with a number of diseases, including depression and anxiety, Parkinson's disease, and orthostatic intolerance [20,37-39], the biogenic amine transporter subfamily is probably the most biomedically interesting subgroup of the NSS family. All members of this subfamily transport common neurotransmitters within the brain, including dopamine, norepinephrine and epinephrine, and serotonin. The first three are all catecholamines (which are synthesized from the amino acid tyrosine), whereas serotonin (5-hydroxytryptoamine) is a derivative of the amino acid tryptophan. ConSequently, while all four are aromatic amines, there is significant chemical diversity discriminating the catecholamines from serotonin. It is again encouraging that this chemical distinction between substrates (Figure 2b) is recapitulated within the subfamily phylogeny (Figure 3b). Functional studies have shown that in general, the catecholamine transporters transport all catecholamine substrates (albeit with differing affinities), whereas they discriminate against serotonin. Conversely, the serotonin transporter favors serotonin and discriminates against catecholamine substrates [40].

There are several significant differences between the two amino acid transporter subfamilies. For example, the first (amino acid #1) is much larger than the second (18 vs. 5 sequences). Further, the first is much better biophysically characterized than subfamily #2; there is only one ORFan within amino acid #1 (which represents 5.5%), whereas three out of five are ORFans within amino acid #2. Within subfamily #1, most of the sequences are experimentally characterized as either glycine or proline transporters, whereas some are simply annotated as amino acid transporters. Across the subfamily, there is only one ORFan, which, based on its out-group consensus, has now been functionally assigned as a glycine transporter. In the smaller subfamily #2, three of the sequences are experimentally annotated as amino acid symporters. The other two, which are diverged from the first three, remain ORFans.

Of the remaining two subfamilies, one is composed of a wide variety of Archaeal and Bacterial symporters; this class includes LeuT_Aa_. We generically designate this subfamily prokaryotic. In fact, this subfamily could be further subdivided into a variety of different out-groups. However, we chose not to further subdivide this portion of the tree due to the fact that there are no conserved functional distinctions present. Nothing else obvious (i.e., organismal taxa, functional annotation, etc.) appears to cluster in the same way as the prokaryotic subfamily. Nevertheless, consistent with the work of Quick et al. [41], there is a mix of eleven and twelve transmembrane homologs within the prokaryotic subfamily, whereas the other subfamilies are always twelve.

The final subfamily, designated as Renal, is generically labeled based on the fact that six of the seven experimentally characterized sequences are found within the kidney and/or intestine. The seventh is found in the brain. The remaining 17 sequences within the subfamily are uncharacterized. Unfortunately, only one of the uncharacterized sequences can be annotated using the phylogenomic approach described above. This is because the experimental annotations are clustered within only two regions of this diverse subfamily. Based on the location of the branch point, we also include the homologs highlighted in dark purple within the Renal system subfamily; however, no experimental support for this prediction is currently available. In addition, the experimental characterizations of the seven proteins are not very informative. Six are generically annotated as "Sodium- and chloride-dependent transporters," while the seventh is a proline/hydroxyproline transporter called the IMINO system.

### Overall assessment of functional site predictions

The previous section clearly demonstrates that observed functional differences within the NSS family are consistent with its phylogeny. This information is important for broadly understanding familial diversity. However, being able to interrogate specific residue differences at functional sites generally provides more insight than interrogations at nonfunctional sites. ConSequently, we also assess the ability of sequence-based methods to accurately predict important sites within the family. In this report, we attempt to predict functional sites using a variety of different bioinformatic approaches. Note that Soyer and Goldstein [42] similarly published an impressive group of functional site predictions within the NSS family using a site class model of evolution. Our report is distinguished from theirs in four ways: (i) they did not have the hindsight of the LeuT_Aa _structure, (ii) we apply a broader array of functional site prediction strategies, which we find provide both orthogonal and complementary results, (iii) we attempt to correlate the evolutionary differences with functional divergence, and (iv) using published mutagenesis studies of the most well-characterized members of the NSS family (serotonin, dopamine, and GABA transporters), we provide extensive evidence supporting the reliability of our bioinformatic approach in predicting functional sites in protein families.

**Figure 4:**
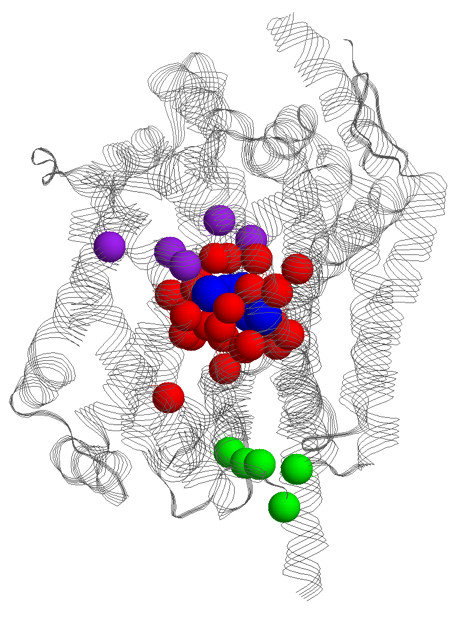
The entire LeuT_Aa _structure is shown with the α-carbons of the 34 known functional sites highlighted; the binding site residues are colored red, cytoplasmic gate residues are colored green, and extracellular/periplasmic gate residues are colored purple. The leucine substrate and sodium ions are rendered in spacefill and colored blue.

From the LeuT_Aa _structure (see Figure 4), a canonical set of 34 functional sites has been identified [10]. The functional sites include the two unwound transmembrane helices (TM1 and TM6) at the leucine-binding site, residues directly involved in substrate binding, two Na^+ ^binding sites, two extended interaction networks at the cytoplasmic and extracellular gates, and one residue (Glu62) that stabilizes the unwound TM6 helix. The 34 functional sites are detailed in Table 2. Here, we apply six different functional site prediction strategies (see Methods for details). The six methods are based on phylogenetic motifs [43], conserved motifs [43], individual site conservation, the Consurf [44] conservation algorithm (which is called Rate4Site [45]), evolutionary trace [46], and prediction of specificity determining positions [47]. Table 3 describes each method's performance on the complete benchmark, whereas Table 4 provides performance assessment across the structurally observed binding sites, the predicted cytoplasmic gate residues and the predicted extracellular/periplasmic gate residues.

**Table 2:** Structural assessment of the functional site predictions.^1^

**LeuT**_**Aa**_	**rGAT1**	**rSERT**	**hDAT**	**Structural location**	**Description**	**Experimental evidence**	**Predicted by**^2^
*(a.) Substrate/Na*^+^*-binding sites*							

G20	**G59**^+^	**G94**^+^	G75	TM1a	Na^+ ^binding site	[2–95]	P,S,R,E
N21	**Y60**^+^	**Y95**^+^	**F76**^+^	TM1a	Leu binding site	[94–99]	None
A22	A61	A96	A77	TM1a	Leu & Na^+ ^binding sites	None	F,R
V23	**I62**^+^	V97	V78	Unwound TM1	Na^+ ^binding site	[95]	F
G24	**G63**^+^	**D98**^+^	**D79**^+^	Unwound TM1	Leu binding site	[95, 97, 100, 101]	F,R,E,D
L25	**L64**^+^	**L99**^+^	**L80**^+^	TM1b	Leu binding site	[92, 95, 102]	P,F,S,R,E
G26	**G65**^+^	**G100**^+^	A81	TM1b	Leu binding site	[92, 95]	P,F,S,R
N27	**N66**^+^	**N101**^+^	N82	TM1b	Na^+ ^binding site	[92, 95]	P,F,S,R,E
E62	**E101**^+^	**E136**^+^	**E117**^+^	TM2	Stabilizes TM6 unwound region	[55–58]	P,F,S,R,E
V104	**L136**^-^	**I173**^+^	**V152**^+^	TM3	Leu binding site	[93, 96, 103–105]	None
Y108	**Y140**^+^	**Y176**^+^	**Y156**^+^	TM3	Leu binding site	[106–108]	F,S,R
F252	F293	F334	**C319**^+^	TM6a	Leu binding site	[109]	P,F,S,R
F253	**F294**^+^	**F335**^+^	**F320**^+^	TM6a	Leu binding site	[93, 98, 103, 110]	P,F,S
T254	**S295**^+^	S336	**S321**^+^	TM6a	Leu & Na^+ ^binding sites	[108, 110, 111]	P,F,E
S256	G297	G338	**G323**^+^	Unwound TM6	Leu binding site	[112]	P,F,E
L257	**L298**^+^	**P339**^+^	V324	Unwound TM6	Leu binding site	[110, 113]	P
G258	G299	G340	**G325**^+^	Unwound TM6	Leu binding site	[98]	P
F259	L300	**F341**^+^	**F326**^-^	Unwound TM6	Leu binding site	[93, 98, 103]	P
G260	G301	G342	**G327**^+^	Unwound TM6	Leu binding site	[112]	P,S,E
N286	**N327**^+^	N368	N353	TM7	Na^+ ^binding site	[111, 114]	P,F,R
A351	L392	L434	L418	TM8	Na^+ ^binding site	None	F,R,E,D
T354	**D395**^+^	D437	**D42**^+^	TM8	Na^+ ^binding site	[111, 115]	P,F,R,E,D
S355	**S396**^+^	**S438**^+^	S422	TM8	Leu & Na^+ ^binding sites	[93, 103, 111]	P,F,S,D
I359	T400	**G442**^+^	G426	TM8	Leu binding site	[93, 103]	None

*(b.) Cytoplasmic gate*							

R5	**R44**^+^	R70	**R60**^+^		Cytoplasmic gate	[116]	S
W8	**W47**^+^	W82	**W63**^+^		Cytoplasmic gate	[58, 116]	S
S267	S308	S349	S334		Cytoplasmic gate	None	S,R,E
Y268	**Y309**^-^	Y350	**Y335**^+^		Cytoplasmic gate	[57, 106, 115]	S,E
D369	D410	D452	**D436**^+^		Cytoplasmic gate	[58, 115]	S,E

*(b.) Extracellular/periplasmic gate*							

R30	**R69**^+^	**R104**^+^	**R85**^+^	TM1b	Extracellular gate	[57, 92, 95, 97, 117]	P,F,S,R,E
Y47	**Y86**^+^	Y121	**Y102**^+^	TM2	Extracellular gate	[106, 108]	P,F,S,R,E
Q250	**Q291**^+^	Q332	**Q317**^+^	TM6a	Extracellular gate	[57, 108, 110]	P,F,S,R,E
E290	**S331**^+^	S372	L355	TM7	Extracellular gate	[111, 114]	P
D404	D451	**E493**^+^	**D476**^+^	TM10	Extracellular gate	[58, 104]	P,S

^1 ^The importance of the functional site benchmark has been clearly established by Yamashita et al. from the LeuT_Aa _structure [10]. Residue numbers correspond to those of LeuT_Aa_, rGAT1, rSERT, and hDAT (columns 1–4, respectively). Residues shown in bold with + are those whose functional role is supported by experimental data. Residues shown in bold with - are those whose functional role is not supported by experimental data. Our literature search failed to find mutagenesis evidence for residues without boldface. The criterion for positive experimental confirmation was a minimum of two-fold reduction in substrate uptake rate and/or affinity of the single site mutant compared to WT.^2 ^Six unique bioinformatic approaches were utilized in order to predict functional sites within the NSS family. The methods employed are: P = phylogenetic motif, F = false positive expectation, S = site conservation, R = Rate4Site, E = evolutionary trace, and D = SDPpred.

**Table 3:** Coverage and accuracy of the various functional site prediction schemes across all 34 functional sites.

**Method**	**Coverage**^1^	**Absolute Accuracy**^2^	**Relative Accuracy**^3^	**Overall Performance**^4^
*(a.) Unique methods*				

Phylogenetic motif	0.62 (21)	0.24 (89)	0.55 (38)	0.40
False positive expectation	0.53 (18)	0.35 (51)	0.90 (20)	0.43
Site conservation	0.59 (20)	0.35 (58)	---	0.45
Rate4Site	0.50 (17)	0.37 (46)	---	0.43
ET	0.44 (15)	0.27 (56)	---	0.34
SDPpred	0.12 (4)	0.27 (15)	---	0.19

*(b.) Hybrid methods*				

Union (PM + FPE)	0.77 (26)	0.22 (116)	0.45 (58)	0.42
Intersect_2^5^	0.71 (24)	0.29 (84)	---	0.46
Intersect_3^5^	0.56 (19)	0.44 (43)	---	0.50
Intersect_4^5^	0.32 (11)	0.50 (22)	---	0.40
Intersect_5^5^	0.18 (6)	0.67 (9)	---	0.37

^1 ^Coverage describes the fraction of correctly predicted functional sites from Table 2; the raw count is provided in parentheses. ^2 ^Absolute accuracy is defined as the ratio of correct predictions to total alignment positions predicted (provided in parentheses). ^3 ^Because the PM, FPE, and Union methods are based on alignment windows (versus individual sites within the alignment), relative accuracy is also calculated. Relative accuracy is similar to absolute accuracy, but describes the ratio of correct predictions to the total number of alignment windows (also provided in parentheses). ^4 ^Overall performance is calculated as the Cartesian distance between (coverage, accuracy) of each method and that of a perfect method (coverage = 1.00, accuracy = 1.00). The distance is normalized such that a method with 0.00 coverage and accuracy would have a value of unity, which is then subtracted from one in order to stay consistent with the coverage and accuracy scales. ^5 ^The Intersect predictions describe a hybrid approach composed of the unique prediction strategies. Whenever the number of predictions for a particular site are greater than the intersect value, that site is put forth as a prediction (e.g., Intersect_2 represents the intersection of any two unique methods). Due to poor performance, the SDPpred method is not considered within the Intersect results.

**Table 4:** Performance of the various functional site prediction schemes across all functional sites.^1^

**Method**	**Binding sites**	**Cytoplasmic gate**	**Extracellular gate**
*(a.) Unique methods*			

Phylogenetic motif	0.68; 0.18; 0.38	0.00; 0.00; 0.00	1.00; 0.06; 0.33
False positive expectation	0.63; 0.29; 0.43	0.00; 0.00; 0.00	0.60; 0.06; 0.28
Site conservation	0.46; 0.19; 0.31	1.00; 0.09; 0.93	0.80; 0.07; 0.33
Rate4Site	0.54; 0.28 0.39	0.20; 0.02; 0.10	0.60; 0.07; 0.28
ET	0.38; 0.16; 0.26	0.60; 0.05; 0.27	0.60; 0.05; 0.27
SDPpred	0.21; 0.56; 0.36	0.00; 0.00; 0.00	0.00; 0.00; 0.00

*(b.) Hybrid methods*			

Union (PM + FPE)	0.88; 0.18; 0.41	0.00; 0.00; 0.00	1.00; 0.04; 0.32
Intersect_2	0.71; 0.20; 0.40	0.60; 0.04; 0.26	0.80; 0.05; 0.31
Intersect_3	0.42; 0.35; 0.38	0.20; 0.02; 0.10	0.60; 0.07; 0.28
Intersect_4	0.33; 0.36; 0.34	0.00; 0.00; 0.00	0.60; 0.14; 0.33
Intersect_5	0.13; 0.33; 0.22	0.00; 0.00; 0.00	0.60; 0.33; 0.45

^1 ^Each cell of the table includes: coverage; accuracy; and overall performance of the method on each subset of the complete benchmark dataset. The three subsets are defined in Table 2.

Using phylogenetic motifs (PMs), many of these functional sites are predicted. PMs, which are a functional site prediction strategy we have developed [43], are sequence alignment regions that mirror the overall familial phylogeny. PMs are calculated using the MINER program [48]. Across a wide array of (mostly globular) proteins, PMs have been shown to consistently correspond to functional sites defined by both surface loops and active site clefts. However, the method has only been sparsely applied to integral membrane proteins. The last column in Table 2 demonstrates that PMs correspond to the majority of the known functional sites. In fact, PM coverage (defined as the number of correct predictions within the benchmark dataset) is the best of the five methods considered. On the other hand, this sensitivity comes at the cost of specificity. The accuracy (defined as the ratio of correct to total alignment positions predicted) of the PM predictions is only 0.24 (see Table 3), which is the lowest of the six methods. Overall, PM predictions are ranked fourth (of six) when both accuracy and coverage are considered (see Table 3 for the definition of overall performance).

Conserved regions (i.e., traditional motifs) are also identified using MINER. MINER identifies these regions based on a calculated False Positive Expectation (FPE) value for each alignment window [43]. Windows with smaller FPEs are more conserved, and thus less likely to be encountered within a database by random chance. Table 3 indicates that the FPE method has a good balance between sensitivity and specificity; however, this balance is only maintained in predictions of the binding sites (see Table 4). Nevertheless, its overall performance is the second best of the methods presented here (see Additional file 3). A cursory analysis of the last column Table 2 suggests that FPE predictions are orthogonal to the PM predictions. It follows that a simple union of the PM and FPE prediction sets predicts 26 of the 34 functional sites. We develop this Union approach because, while there is frequently overlap within the PM and FPE results [43,49], they are fundamentally based on two distinct (albeit related) phenomena. PMs are based on phylogenetic topology, whereas FPE is based on sequence conservation. The coverage of the Union approach is extremely good. In fact, it predicts all nine Na^+ ^binding sites and ten of twelve leucine-binding site residues.

Four additional prediction techniques are considered: site conservation, Rate4Site [45], evolutionary trace [46], and predictions of specificity determining positions [47]. The site conservation (SC85) approach simply returns positions that are conserved greater than 85% within the multiple sequence alignment. While simplistic, this approach can be quite powerful (see Table 3). In fact, it is determined to have the best overall performance of any method considered. Interestingly, SC85 does very well on predictions of the cytoplasmic and extracellular/periplasmic gate residues (coverage = 1.00 and 0.80, respectively). Its coverage of the binding site residues is 0.46.

Akin to SC85 is Rate4Site, which attempts to describe the relative evolutionary rate at each position within the alignment using either Bayesian [50] or Maximum Likelihood [45] statistics. In both, phylogenetic tree topology and stochastic evolutionary considerations are considered. Impressively, the accuracy of Rate4Site, which is the best of the five methods, is 0.37 (Table 3). It tied for second in overall performance. When considering the benchmark subsets, Rate4Site does better than SC85 on the binding site residues, but does much poorer on the two gate subsets.

The last two methods analyzed both attempt to predict alignment positions that define subfamily specificity. Evolutionary trace (ET) is one of the most widely used methods to predict protein functional sites [51,52]. The approach looks for positions that are conserved within out-groups on an input phylogenetic tree [46]. The standard application of the approach is to then map both *trace residues *and conserved positions to a representative structure. However, ET predictions without structure can also provide good functional site predictions [53]. Numerous reports have shown that the approach does a very good job of identifying known functional sites; see Lichtarge et al. for a recent review [54]. In an analogous way, SDPpred (Specificity Determining Position prediction) uses mutual information to identify alignment positions where the amino acid distribution is closely associated with the functional specificities. Here, both are the two worst performing methods considered overall; the overall performance of ET and SDPpred is 0.35 and 0.19, respectively. Nevertheless, ET does have respectable coverage on both of the gate subsets (0.60 in both cases); its coverage of the binding site subset is only 0.38. The overall coverage (0.12) of the SDPpred method is very poor; however, due to so few predictions, its accuracy is reasonable (0.27).

Taken together, the functional site predictions are quite impressive. All but three of the functional sites are predicted by at least one method. There is also appreciable overlap within the predictions. Across the five unique methods, 20 of the 34 functional sites are predicted by at least three different techniques; four more are predicted by two methods. Additional file 4 details the results from prediction methods across the entire LeuT_Aa _sequence. Except for SDPpred, the coverage of each unique method is always better than 0.44 (Table 3). However, consistent with earlier investigations [43], the coverage by ET is slightly poorer than those of other approaches. Impressively, the coverage by Union is 0.77. The absolute accuracy is best for Rate4Site (0.37); however, FPE and SC85 are very close (both are 0.035). This, of course, makes sense as all three methods highlight sequence conservation. Curiously, as demonstrated below, there is little overlap between the FPE and SC85 predictions.

While the coverage of the PM approach is the best, the absolute accuracy of the PM method (and consequently, the Union method) is substantially lower than the others. Note that it could be argued that simply comparing absolute accuracy is not a fair metric since PM, FPE, and Union are based on alignment *windows*. SC85, Rate4Site, and ET predictions are based on single alignment positions, whereas the window-based prediction identifies five sites at a time, meaning the denominator of the accuracy ratio is increased. As such, we introduce relative accuracy, which is defined as the number of correct predictions/total number of alignment windows predicted (Table 3). The relative accuracies of these three approaches are substantially higher than all absolute accuracies. We do not want to overemphasize these results as, again, this is not a perfect comparison – one should never make too much of comparisons of apples and oranges! Nevertheless, the absolute and relative accuracies provide an accuracy range that is in qualitative agreement with the other approaches.

### Predictions of specific functional site sets

**Figure 5:**
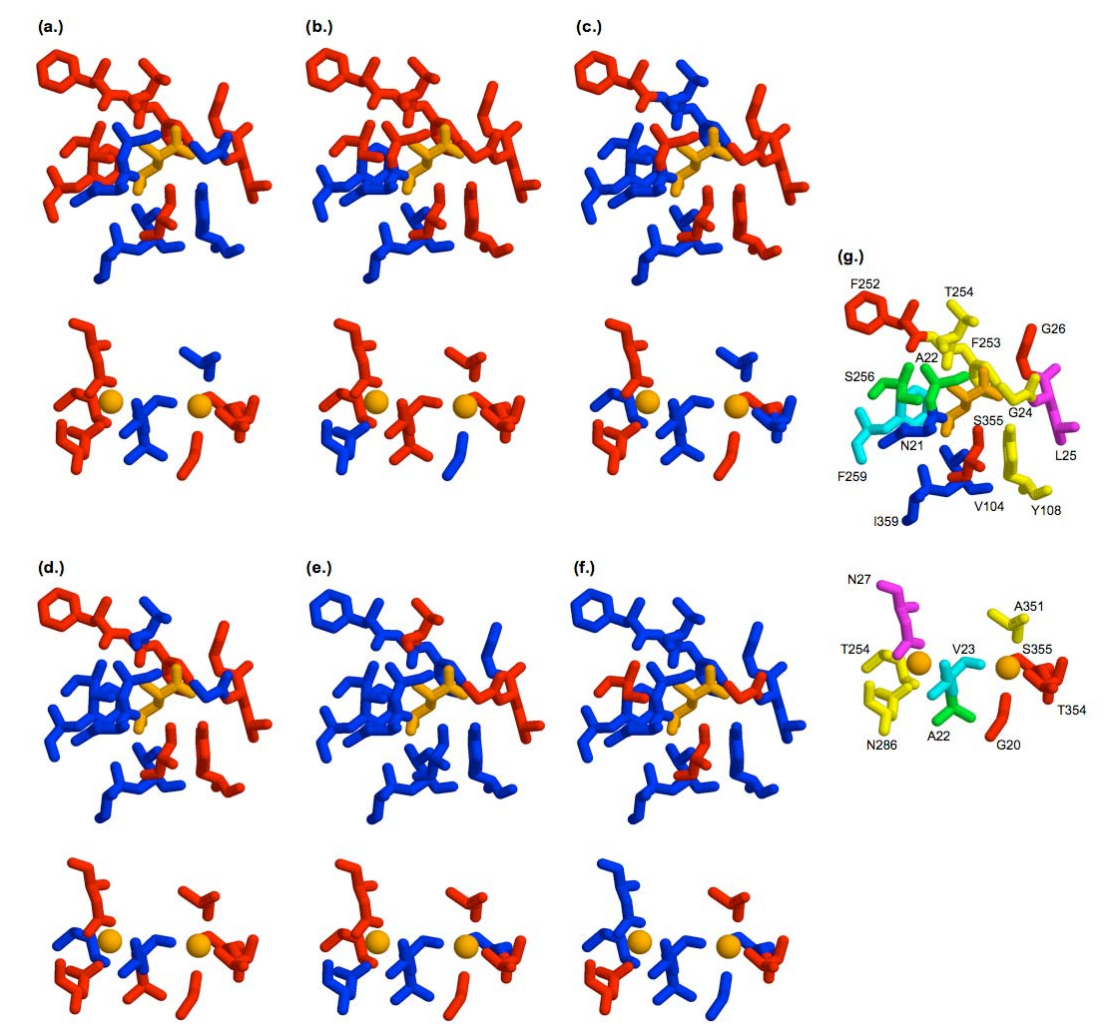
Structural descriptions of the functional site predictions within the leucine (top) and sodium ion (bottom) binding sites. Red indicates functional residues that are predicted, whereas blue indicates not predicted by **(a) **phylogenetic motifs, **(b) **false positive expectation, **(c) **site conservation, **(d) **Rate4Site, **(e) **evolutionary trace, and **(f) **SDPpred. **(g) **In the last frame, residues are color-coded based on the number of methods (excluding SDPpred) that predict each position (0 = blue, 1 = cyan, 2 = green, 3 = yellow, 4 = red, and 5 = magenta). In all cases, the leucine and sodium ion substrates are colored orange.

The leucine substrate, two sodium ions, and a chloride ion are co-crystallized within the LeuT_Aa _structure. The biological importance of the leucine and sodium ion binding sites is unambiguous, thus it follows that knowing how well the methods predict the leucine and sodium ion binding sites is imperative to their assessment. (Note that the significance of the chloride ion-binding site, which is structurally remote from the others, is still being debated, thus it is omitted from this analysis.) Within the functional site benchmark, 14 residues are defined as part of the leucine-binding site, whereas nine constitute the sodium ion binding sites (see Table 2). Three residues (Ala22, Thr254, and Ser355) are involved in both. Figure 5 clearly indicates that the five different methods result in substantially different predictions. Interestingly, the two methods based on sequence windows (PM and FPE) have better coverage of these residues (14 and 16, respectively). While it is straightforward to view their increased coverage as a simple fact that they predict sequence chunks, this is not the case. In fact, the total number of alignment positions predicted by FPE is less than SC85 and ET. The other three methods (SC85, Rate4Site, and ET) predict 11, 14, and 9, respectively. The poor coverage of the leucine-binding site by ET and SDPpred, both of which look (at least in part) for subfamily specific residues, is particularly notable. The good coverage of the Leucine-binding site by the remaining conservation measures suggests that the general binding site location is conserved across the family; however, results from the class specific methods (ET and SDPpred) suggest that the exact details of the transporter-substrate interaction are likely defined by a different set of residue positions across the family. Figure 5g color-codes the binding site residues by the number of different methods that predict them. Encouragingly, 71% of the binding site residues are predicted by at least two different methods, and 59% of the binding sites are predicted by at least three different methods. Coverage of the binding site and extracellular/periplasmic gate residues is also quite good. The coverage of each by three or more methods is 67% and 60%. Only one (10%) of the cytoplasmic gate residues is predicted by three or more methods.

Across the canonical set of functional sites, all but three (Asn21, Val104, and Ile359) are predicted by at least one method. All three are part of the leucine-binding site. Of these three, none is conserved greater than 35%, indicating a lack of strong evolutionary pressure acting on these positions. Curiously, all three make van der Waals contacts with the leucine substrate. Particularly interesting are Val104 and Ile359. With just a handful of exceptions, both positions are chemically conserved; the positions are almost exclusively hydrophobic. This result makes sense within the context of hydrophobic amino acid and biogenic amine transporters (see Figure 2b) as both groups of substrates are amphipathic with large nonpolar regions that could interact with the hydrophobic binding site residues – they form the base of the binding site pocket around the leucine sidechain (see Additional file 5). However, it is not clear how nonpolar residues at these two positions can interact with the osmolytes or polar amino acids, both of which have charged groups on both ends of the substrate. This ambiguity is presented to highlight the more gratuitous shortcomings of the sequence-based approaches employed here, and will likely have to wait for further structural studies to be resolved. In spite of the inability of the methods to predict these two sites, all five methods predict sites proximal (within five alignment positions) to Ile359, and three of the methods (all but ET and PM) do the same for Val104.

The extracellular/periplasmic gate residues, which are proximal to the leucine/Na^+ ^binding sites, are generally predicted well by SC85 and PMs, which correctly predict all five and four positions, respectively. FPE, Rate4Site, and ET predict three of the five. Conversely, there is more diversity within the cytoplasmic gate residue predictions. SC85 predicts all five, whereas PMs and FPE fail to predict any; the other methods fall somewhere in between. SDPpred fails to predict any cytoplasmic or extracellular/periplasmic gate residues. When considering the unwound transmembrane helices, complementarity between prediction schemes is similarly observed. Only FPE is able to predict the unwound regions of TM1, whereas only PMs predict all five unwound residues in TM6. Glu62 is thought to stabilize the unwound region in TM6. All five methods predict Glu62 to be functional. Remarkably, mutation of equivalent residues in at all three eukaryotic homologs (rSERT, hDAT, and rGAT1) confirms that this position is important [55-58]. Additionally, mutagenesis experiments reinforce the importance of the cytoplasmic gate residues, extracellular/periplasmic gate residues, and the leucine/Na^+ ^binding sites (see Table 2).

### Complementarity and overlap within the predictions

As alluded to above, an important conclusion from this work relates to the high degree of complementarity between the different prediction methods. A cursory analysis of the results clearly suggests that the methods provide different prediction sets. Complementary between each pair of prediction sets is described by vector orthogonality (see Table 5). Orthogonality is calculated as the Euclidean distance between a vector pair representing two different functional site prediction sets. Each vector has 34 dimensions (corresponding to the 34 functional sites identified in Table 2). Each dimension is simply a binary possibility (1 = correctly predicted functional site; 0 = missed functional site). The values are then adjusted such that two completely orthogonal sets have a score of 1.0, whereas completely identical sets would have a score 0.0. The FPE/Rate4Site pair is the most similar; the pair has 26 (out of 34 possible) matches when comparing their prediction sets. After excluding SDPpred, which predicts far few functional sites than the other methods, it is surprising that methods ostensibly based on the same approach (phylogeny in the case of the PM and ET pair and conservation in the case of FPE and SC85 pair) are the most dissimilar – they each have 16 mismatches. Nevertheless, the extent of orthogonality is fairly consistent across all possible pairs, the average and standard deviation is 0.66 and 0.09, respectively.

**Table 5:** Complementarity within the six unique functional site prediction schemes.^1^

	**PM**	**FPE**	**SC85**	**Rate4Site**	**ET**	**SDPpred**
**PM**	---	---	---	---	---	---
**FPE**	0.62	---	---	---	---	---
**SC85**	0.62	0.69	---	---	---	---
**Rate4Site**	0.66	0.49	0.59	---	---	---
**ET**	0.69	0.64	0.62	0.62	---	---
**SDPpred**	0.80	0.66	0.86	0.71	0.64	---

^1 ^Complementarity is calculated as the Euclidean distance between each vector pair describing the results within Table 2, meaning each vector has 34 dimensions. Each dimension is simply a binary possibility (1 = predicted functional site; 0 = not predicted functional site). The values reported are normalized such that two completely orthogonal vectors will have a distance of one; two vectors that are absolutely the same will have a distance of 0.00, whereas two completely orthogonal vectors will have a distance of 1.00. The average value and standard deviation across the matrix are 0.66 and 0.09, respectively, which corresponds to 15.0 and 4.1 differences.

**Figure 6:**
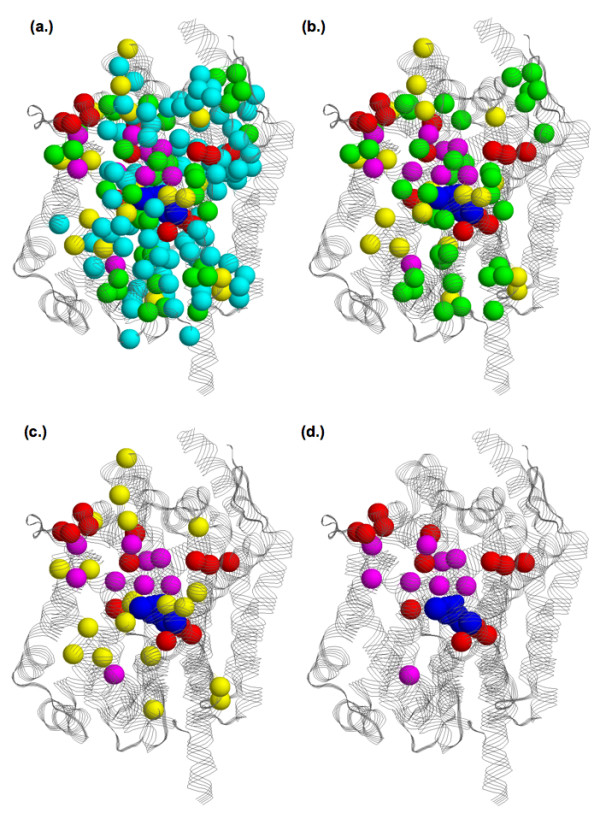
Structural superposition of all functional site predictions onto the LeuT_Aa _structure. Spheres represent α-carbons of the predicted residues, which are color-coded by the number of methods (excluding SDPpred) that predict each residue (1 = cyan, 2 = green, 3 = yellow, 4 = red, and 5 = magenta). The four views show sites predicted by at least **(a) **one, **(b) **two, **(c) **three, and **(d) **four methods. In all cases, the leucine, sodium ions, and chloride ion are colored blue.

We also investigate how predictions based on simple intersections of the various unique methods improve prediction accuracy. Meaning, only positions that are simultaneously predicted by multiple methods are put forth as a prediction. Due to poor overall performance, SDPpred is excluded from this analysis. Moreover, SDPpred only predicts positions that are predicted by at least three other unique methods. Table 3 demonstrates that the simple Intersect method clearly improves performance. Only nine positions are concurrently predicted by all five schemes. As discussed above, one corresponds to Glu62; three others correspond to binding site residues; and three correspond to extracellular/periplasmic gate residues. When a site is predicted by any four methods, 22 are predicted, half of which are included in the functional site set. Impressively, relaxing the criterion to any three methods raises the coverage and accuracy to 0.56 and 0.44, respectively. When any two methods intersect, the accuracy is reduced to 0.29 (which is within the range of the individual methods), but the coverage increases to an impressive 0.71. Interestingly, Figure 6 indicates that predictions with better support, meaning they are predicted by multiple methods, are more likely to cluster around the leucine-binding site and the proposed transport route (discussed below). It will be quite interesting to determine from future investigations if the Intersect predictions (vs. individual methods) do a better job of predicting positions that exhibit a functionally deleterious phenotype upon mutation.

### Substrate transport route

While the unwound regions of TM1 and TM6 clearly constitute the leucine-binding site within LeuT_Aa_, Yamashita et al. also predict that they act as joints within the protein structure, which allow it to change conformations (outward facing open ↔ closed ↔ inward facing open) during substrate transport. This model is consistent with the general alternating access model proposed for carriers [5,7,24,59]. Their model predicts that substrate passage occurs along a route defined by TM1, TM3, TM6, TM8 and TM10. The solved LeuT_Aa _structure is thought to be in the closed or occluded conformation. The Yamashita model proposes that the salt bridge between Arg30 and Asp404 (both of which are included in our 34 functional sites) is broken when the structure occupies the outward facing open conformation. Conformational changes within the extracellular helices EL2 and EL4 are believed to concomitantly occur on loss of the salt bridge. Their model is supported by experimental data showing that EL2 and EL4 adjust during transport [60,61]. More drastic conformational changes are believed to occur on opening of the cytoplasmic gate. On loss of a second "locking" salt bridge, this time between Arg5 and Asp369 (both of which are also included in our 34 functional sites), TM1a and TM6b are believed to "swing out" via the hinges provided by the unwound helices. This conformational change at the cytoplasmic gate provides an opening for the substrate and accompanying ions to dissociate into the cytoplasm, thus completing ion/substrate cotranslocation across the plasma membrane. Intriguingly, the structure suggests that the two Na^+ ^ions and the leucine substrate share the same permeation pathway.

**Figure 7:**
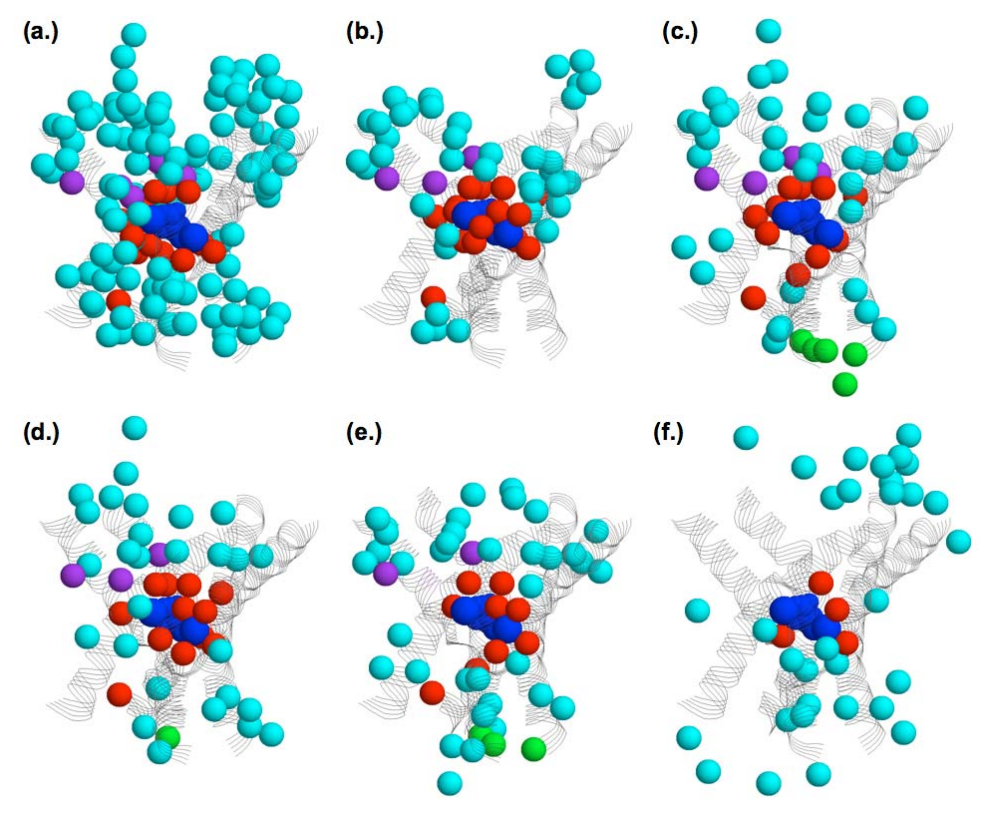
Structural superposition of the functional site benchmark and predictions onto the LeuT_Aa _structure. The proposed substrate transport route (defined by TM1, TM3, TM6, TM8 and TM10) and the functional site predictions from each method are displayed in the same orientation as Figure 2. The six methods are: **(a) **phylogenetic motifs, **(b) **false positive expectation, **(c) **site conservation, **(d) **Rate4Site, **(e) **evolutionary trace, and **(f) **SDPpred. Coloring of the 34 functional sites is the same as Figure 2; cyan indicates predictions not matching any of the functional sites.

**Figure 8:**
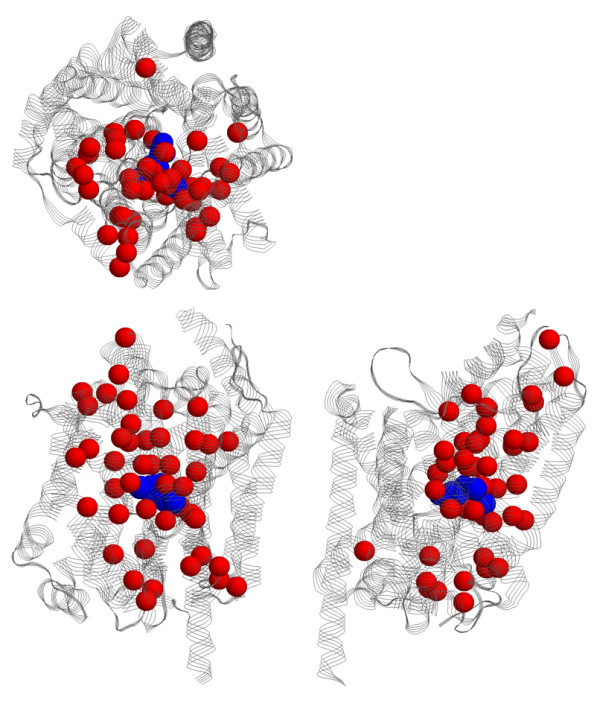
Three orthogonal views of the Rate4Site predictions superimposed onto the LeuT_Aa _structure. Red spheres correspond to α-carbons of the predicted residues. As can be clearly seen, the predictions cluster along the proposed transport route. Similar results are observed in the other methods as well. The orientation on the lower-left is the same as in Figure 6a. The view on the upper-left is a rotation of 90° in the x-direction, whereas the view on the lower-right is a rotation of 90° in the y-direction.

Figure 7, which is the same orientation and coloring scheme of Figure 4, shows that the known functional sites and the functional site predictions primarily cluster along the proposed ion/substrate permeation pathway. This result strongly supports the model put forth by Yamashita et al. Note that only TM1, TM3, TM6, TM8 and TM10 are shown in Figure 7. Many of the functional site predictions correspond to these regions. However, there are also predictions that do not explicitly correspond to these five transmembrane helices. Nevertheless, the functional site predictions tend to structurally cluster near the proposed permeation pathway. The only exception to this scenario is with SDPpred, whose predictions are commonly removed from the proposed transport route (see Figure 7f). Similarly, as discussed above, highly supported predictions (meaning positions predicted by three or more techniques) cluster better along the proposed permeation route than by predictions only identified by one or two techniques (Figure 6). The clustering of the functional site predictions along the proposed passage route is better established in Figure 8, which displays the Rate4Site predictions within three orthogonal views. Similar clustering is observed in the other prediction schemes as well.

### Assessment of functional sites from available mutation data

Since cloning of the mammalian members of the NSS family of transporters in the early 1990s [62-68], several laboratories have devoted considerable effort to understanding the structure-function relationships of these proteins. In particular, site-directed mutagenesis has served as a powerful tool with which to identify amino acid residues that govern various aspects of transporter function (e.g., substrate binding, substrate affinities, turnover rate, etc.). In this report, we have used representative results gained from these functional studies in order to provide additional support for the 34 structurally derived functional sites. Note that this list is not meant to be exhaustive; rather, it is simply meant to support or refute the 34 sites examined above. For this purpose, we have chosen representative studies on the rat isoform of the serotonin transporter (rSERT), human dopamine transporter (hDAT), and rat GABA transporter 1 (rGAT1). These transporters are chosen due to the large number of experimental studies available. As a general rule, the functional data strongly support the data concluded by structural as well as bioinformatic approaches (see Table 2). Of the 34 functional sites present, we identify corroborating mutagenesis data in all but three sites (Ala22, Ser267, and Ala351).

The importance of several of the positions within Table 2 is supported by mutagenesis on multiple homologs. For example, seven positions (Asn21, Gly24, Leu25, Arg30, Glu62, Tyr108, and Phe253 using LeuT_Aa _numbering) have experimental mutants with functional phenotypes in all three transporters investigated here (rGAT1, rSERT, and hDAT). Curiously, residue identity is not conserved within two of these positions (Asn21 and Gly24), suggesting that these positions are important to the family's functional diversity. An additional thirteen positions are supported by experimental data in two of the homologs surveyed. Moreover, the general importance in other symporters of each functional class discussed (cytoplasmic gate, extracellular gate, Na^+^-binding site, leucine-binding site, and unwound helix) within the LeuT_Aa _structural description is confirmed [10]. Only three experimentally characterized mutants lack a phenotype; however, in each case, mutation at the equivalent position (Val104, Phe259, and Tyr268) within one of the other two investigated homologs does.

## Conclusion

The results presented herein, using the NSS family as a model system, clearly demonstrate that common bioinformatic methods are able to provide key functional insight into membrane integral proteins in the absence of structural considerations. In general, the methods are able to both predict known functional sites and reveal clear evolutionary discrimination between observed functional distinctions. As such, our results (assuming they can be generalized to other membrane integral families) clearly establish that the employed methods represent a viable means to guide experimental mutagenesis studies and better illuminate functional roles within membrane integral proteins.

More specifically, the phylogeny reveals six clear subfamilies that reflect known functional divisions across the family. In addition, the phylogeny impressively recapitulates several nuanced functional difference within well-characterized subfamilies (i.e., the presence/absence of substrate cross-specificity within GABA symporters and specificity differences between the catecholamine and serotonin symporters). Likewise, functional sites predicted from sequence reproduce most of the canonical set from Yamashita et al. While the methods provide different prediction sets, accuracy is substantially improved when residues are predicted by multiple methods. The observation that the collective set of bioinformatic predictions map to the previously proposed substrate transport route is particularly compelling. Finally, the generality within the importance of these sites across more biomedically relevant members of the NSS family is established by comparing to the large body of experimental mutagenesis results available within the literature.

It is remarkable that even in the absence of high-resolution structural data, earlier mutagenesis experiments and the sequence-based functional site predictions presented here provide sound insight into the role of various amino acid residues in protein function. However, even with the wealth of data now available regarding NSS sequence/structure/function relationships, many questions remain unanswered. For example, even though cotransport of Na^+^/leucine is not Cl^-^-coupled within LeuT_Aa_, the structure revealed a Cl^- ^binding site facing the extracellular space [10]. This is very interesting because Cl^- ^translocation is coupled to Na+/substrate cotransport in some members of the NSS family [69]. A second unanswered question stems from the fact that the LeuT_Aa _structure fails to provide insight into the diversity of functional features (e.g., ion channel versus transporter modes of function) observed in some members of the NSS family [70-72]. However, it is not surprising that these differences are not predicted by the LeuT_Aa _structure since it exhibits only ≤ 25% sequence identity to its mammalian counterparts. Moreover, the functional analysis by Yamashita et al. [10] is insufficient to address such nuanced functional issues (i.e., channel vs. transporter mode) within LeuT_Aa_, meaning that they will have to be addressed experimentally in the future. It is expected that further combinations of structural studies, phylogenomics, advanced functional site prediction techniques, and mutagenesis experiments should provide investigators with a more refined strategy to examine motifs within the larger NSS family and, in particular, within subfamilies where unique functional features exist.

## Methods

### Sequence dataset

Special care was taken to ensure the quality of the sequence dataset used here. The bulk of the dataset was obtained from SwissProt [73], which is rich in eukaryotic homologs. In order to increase the number of bacterial and archaeal sequences, COG0733 (from the COG database [74]) was added to the dataset. The LeuT_Aa _sequence was also added to facilitate structural assessment of the sequence-based predictions. All sequences lacking the primary PROSITE [75] definition of the family (W- [RK]-F- [GPA]- [YF]-x(4)- [NYHS]-G-G- [GCA]-x- [FY]) were purged. PROSITE also contains a secondary definition of the family. However, it is quite divergent within bacterial and archaeal proteins, so it is ignored. All sequence fragments less than 50% of the average length have also been purged. Multiple sequence alignment, using MUSCLE [76,77], followed by phylogenetic analysis (see below) reveals six major subfamilies with a small number (<10) of outliers. The outliers (one of which is an acetylcholine symporter; the others are ORFans) were also purged due to lack of support. The final dataset contains 181 NSS sequences, which have been realigned using MUSCLE; the raw MUSCLE alignment file (.fsa format) is provided in Additional file 6. The results presented above solely use this alignment. Comparison (results not shown) to the recent report by Beuming et al. indicates that our alignment based solely on sequence information is inline with their structurally-informed alignment [78]. Improved multiple sequence alignment strategies (e.g., Mafft [79], Probalign [80], and Probcons [81]) result in very similar alignments (see Additional file 7). Moreover, with the slight exception of the PM method, the results presented herein are largely insensitive to the alignment differences (see Additional file 7). For convenience, all residue numbering corresponds to the LeuT_Aa _structure.

### Phylogenetic analysis

Figure 2 shows the phylogenetic tree calculated on the NSS dataset; the raw phylogenetic tree file (.ph format) is provided in Additional file 1. The tree was generated using the Neighbor-Joining (NJ) phylogenetic reconstruction method implemented in ClustalW [28]. The ClustalW phylogeny is shown here, versus more common approaches like PHYLIP [29], because it is also used within the calculation of phylogenetic motifs (see below). A comparison of the NJ trees computed by ClustalW and PHYLIP (calculated using the Protdist/Neighbor pair of programs) revealed no major differences. In fact, all branches clustered into the same six subfamilies in both trees. Some minor topological differences do exist; however, they are insignificant as the main aim here is to discriminate between subfamilies, and not to provide a robust evolutionary history. Bootstrapping (1000 resamples each) was done on each phylogeny using either ClustalW (see Additional file 2) or PHYLIP. PHYLIP bootstrap results were calculated using the Seqboot/Consence pair of programs.

### Phylogenetic motifs

Phylogenetic motifs (PMs) are sequence alignment fragments that mirror the overall familial phylogeny. In several recent reports [43,49,82-84], we have demonstrated that PMs represent very good functional site predictions from sequence. PMs were calculated using MINER [48], which is now available at [85]. Briefly, MINER uses a sliding sequence window algorithm to comprehensively evaluate the phylogenetic similarity between each window and the complete alignment. An input alignment, is parsed into a series of windows of width = 5, which we have previously demonstrated to be the most sensitive for identifying functional regions [43]. A phylogenetic tree is built for each alignment fragment, and similarity between the window and complete familial tree is quantified using a modified partition metric algorithm [84] that counts the number of topological differences between the two trees, meaning smaller values indicate increased similarity.

The alignment is masked prior to calculating trees on each alignment fragment, meaning that highly gapped positions (>50% gaps) are excluded. Subsequently, phylogenetic trees are calculated using the NJ algorithm within ClustalW. Due to the number of tree calculations required, distance-based trees are used to ensure computational efficiency. For example, in the case of the NSS family, over 580 different trees must be calculated. While we have implemented a parsimony-based version of MINER, the added computational expense is not worth the relatively modest increase in performance for families of this level of divergence [84]. Phylogenetic similarity is quantified using z-scores calculated from the raw partition metric distribution. After all tree comparisons are made, the PSZ threshold can be adjusted to alter what constitutes a "hit". The threshold can be raised or lowered to be more accommodating or stringent, respectively. Our original PM report [43] suggests that PSZ thresholds between -1.0 and -2.0 are ideal. To facilitate large-scale analyses, we have subsequently developed an automated threshold determination algorithm [82]. However, the automated threshold seems to be too stringent in this case. Here, a PSZ threshold of -1.4 is used, which provides sufficient discrimination between signal and noise (Additional file 8). All overlapping windows scoring below the PSZ threshold are defined to be a single PM.

### Motif identification

Traditional motifs (low sequence entropy regions) are also identified by MINER using the False Positive Expectation (FPE) approach described in La et al. [43]. FPE is calculated from the same sequence windows as the PMs; FPE describes the probability of encountering each sequence window randomly. The method describes each window by a regular expression; the overall FPE for each window is calculated as the product probability of the regular expression. For example, the FPE of the regular expression A [V,I,L]T [K,R]P is calculated by the equation: FPE = p(A)·[p(V)+p(I)+p(L)]·p(T)·[p(K)+p(R)]·p(P). Background probabilities are calculated from the COG database [74]. Although not theoretically rigorous, gaps are treated as a 21^st ^residue type. To eliminate over biasing gap probabilities, positions with more than 50% gaps are not tabulated when determining background probabilities. While simplistic, the approach is computationally fast and the results can be compared directly to the observed PMs (see Additional file 8). Moreover, we have demonstrated that FPE results compare very well to MEME results [43].

### Position-specific conservation measures

SC85 simply returns all positions within the alignment that are conserved more than 85%. While simplistic, Tables 3 and 4 clearly demonstrate the utility of the approach. In fact, the method is determined to overall perform the best of the five methods considered. Using a conservation threshold of 100%, none of the residues within the functional site benchmark are predicted. Relaxing the conservation threshold from 100% to 85% results in increased coverage. Relaxing the threshold beyond 85% does not result in improved coverage; however, it does reduce the accuracy (see Additional file 9).

The ConSeq server [86] is used as an alternate conservation scheme [87]. In ConSeq, the evolutionary rate at each alignment position is calculated using Bayesian or maximum likelihood statistics (here, we utilize the Bayesian implementation) using the Rate4Site algorithm. ConSeq reports both normalized conservation scores (whose average and standard deviation are zero and one, respectively) and coarse-grained values range from 1 to 9. Throughout the text, only conservation scores equal to 9 are discussed. While the specificity is reduced, the coverage at level 8 is extremely good; it is better than 80% (see Additional file 9). In fact, the overall performance at level 8 is exactly the same as SC85. However, its accuracy is greatly diminished from level 9 (0.37 to 0.25), which is why we focus on the level 9 results.

### Predictions of class-specific residues

All ET predictions are made using the Evolutionary Trace Server, called TraceSuiteII [88], which is a commonly used web-implementation of the approach [89]. Starting with an input alignment, the Evolutionary Trace Server uses PHYLIP to build a phylogenetic tree. Twenty tree partitions (cut levels), which define the out-groups, are examined. As is done normally, both class specific and conserved sites are listed as functional site predictions. Throughout the text, cut level = 12 is discussed since it provides the best balance between coverage and accuracy (see Additional file 9).

SDPpred [90] is also used to predict class-specific residues [91]. SDPpred requires an alignment and class definitions as input; the six subfamilies discussed above are used to define the classes. The algorithm uses an approach based on mutual information to positions where the amino acid distribution is consistent with the class differences. SDPpred also returns a statistical significance (in the form of z-scores). Throughout the text, all predictions with z-scores greater than 6 are considered; however, other values have little overall affect on the results (see Additional file 9).

## Abbreviations

ABC, ATP binding cassette; NSS, neurotransmitter/sodium symport; LeuT_Aa_, leucine amino acid transporter; GABA, γ-aminobutyric acid; TM, transmembrane; PM, phylogenetic motif; FPE, false positive expectation; SC85, 85% site conservation; ET, evolutionary trace; SDPpred, specificity determining position prediction; rSERT, rat serotonin transporter; hDAT, human dopamine transporter; rGAT1, rat GABA transporter 1.

## Competing interests

The author(s) declares that there are no competing interests. 

## Authors' contributions

DRL and PDK were primarily responsible for execution of the work described herein. SE and UR also contributed to execution of the work and the research design. DRL oversaw the research and wrote the manuscript. All authors have read and approved the final version of the manuscript.

## Supplementary Material

Additional file 1 Phylogenetic tree file. This file contains the raw phylogenetic tree file. Click here for file

Additional file 2 Phylogenetic tree supplementary material. This file contains the phylogenetic tree with bootstrap scores and a list of all experimentally annotated and unannotated leaves. Click here for file

Additional file 3 Prediction method performance figure. This file contains a plot of accuracy vs. coverage for each prediction method considered. Overall performance values are also provided. Click here for file

Additional file 4 Prediction summary figure. This file annotates the LeuT_Aa _sequence with the predictions of each unique method. The sequence is color-coded based on the Intersect results. Click here for file

Additional file 5 Leucine-binding pocket figure. This file contains a figure showing the leucine substrate and the three binding pocked residues that are not predicted by any method used herein. Click here for file

Additional file 6 Muscle alignment file. This file contains the raw Muscle alignment. Click here for file

Additional file 7 Alignment supplementary material. This file contains the sum-of-pairs scores for each alignment pair and a series of tables that detail the predictions of each method as a function of alignment input. Click here for file

Additional file 8 MINER supplemental figures. This file contains supplementary figures associated with MINER. Click here for file

Additional file 9 Prediction threshold summary. This file contains descriptions of performance as a function of threshold level. Click here for file
