# Supplementary material for: Assessing the ability of sequence-based methods to provide functional insight within membrane integral proteins: a case study analyzing the neurotransmitter/Na+ symporter family
Source: BMC Bioinformatics. 2007 Oct 17;8:397. doi: 10.1186/1471-2105-8-397 (PMC2194793; doi:10.1186/1471-2105-8-397)
Supplement: Additional file 2 — Phylogenetic tree supplementary material. This file contains the phylogenetic tree with bootstrap scores and a list of all experimentally annotated and unannotated leaves. [file 1471-2105-8-397-S2.pdf]

Bootstrap results. The ClustalW phylogenetic tree is annotated with the bootstrap results (1000 resamples). The most important result of this analysis is that the major bipartitions discussed in the text are fairly robust. The highlighted bipartitions divide: prokaryotic from rest (green), amino acid #2 from rest (magenta), Renal system from rest (red), amino acid #1 from rest (orange), and osymolytes from biogenic amines (cyan). Similar results are observed within the PHYLIP tree (not shown).

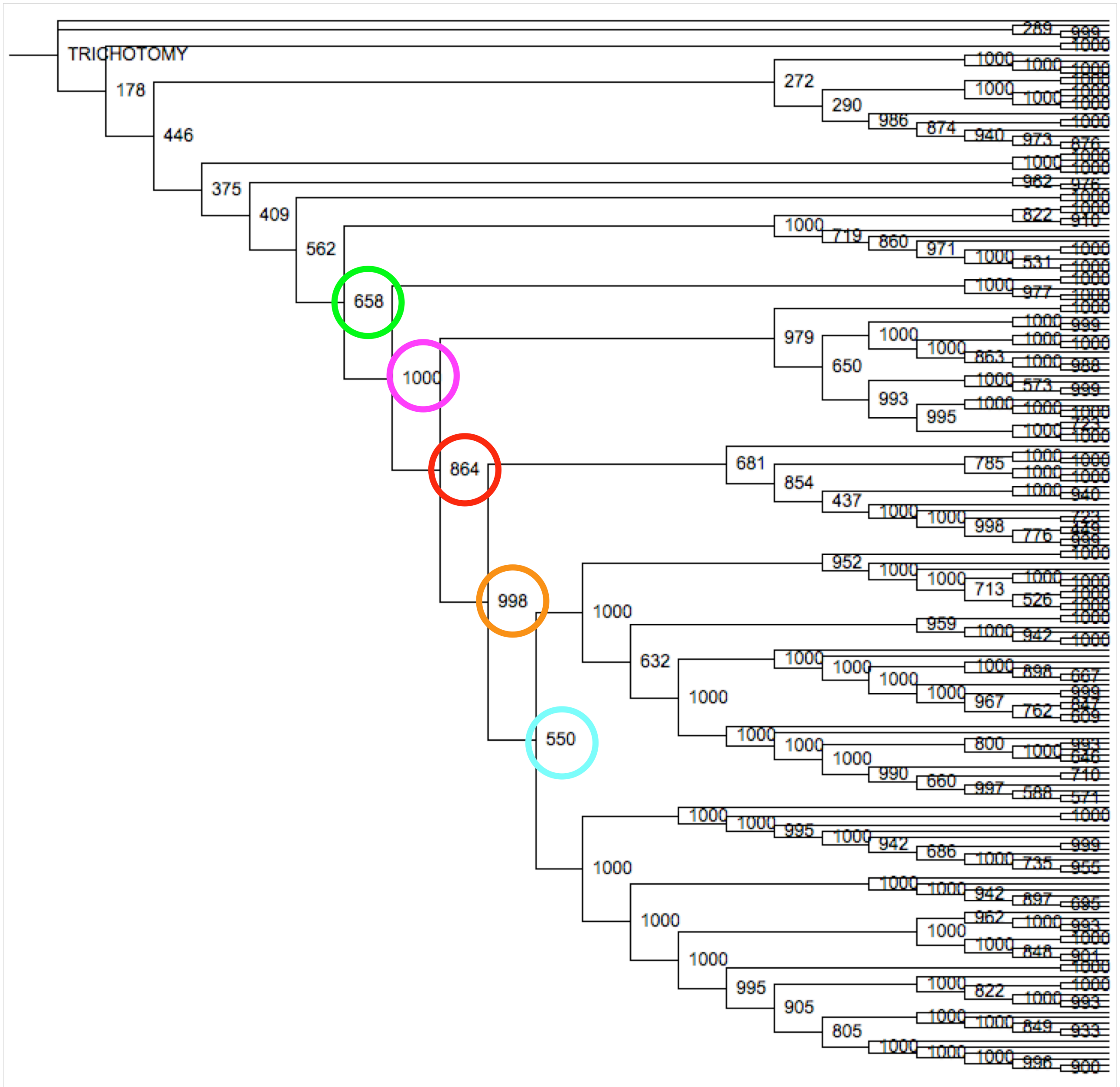

Experimentally annotated vs. unannotated sequences. Sequence labels for each sequence within the alignment are listed from the parent database (either Swiss-Prot/Tremble or COGdb). The twelve labels in bold correspond to the ORFans now assigned to a functional class.

Subfamily	Experimentally annotated	ORFan
Osmolytes (46)	Q91502, O18875, P31661, P48029, Q8VBW1, P28570, O73771, Q91494, Q8BQN8, P30531, Q8N4K8, P48057, P31648, P23978, Q6PCX2, Q9MZ34, O35316, P31643, Q00589, P31641, Q9IBH6, Q8JHX8, Q9DEI6, P48055, P48056, P31651, Q8VCS9, P48065, P27799, Q91503, P48066, P31647, P31650, Q8BKG0, Q8BWA7, P31649, P31646, Q9NSD5, Q8TCC2	**Q68ES1**, **Q5U3E5**, **Q5ISM3**, **Q5F431**, Q801U6, Q801U7, **Q6PA82**
Biogenic amines (44)	Q03614, Q86MC4, Q9V7R0, Q9NB97, O42482, Q9DGN5, P51143, Q9MYW7, Q9MYW8, P23975, Q6NZB4, O55192, Q8R2I2, Q9WTR4, Q63380, Q9WTR3, Q90ZV1, P27922, P23977, Q9R0X6, Q9JJ41, Q61327, Q01959, Q7JHP7, Q9GJT7, Q9GJT6, Q7JHP9, Q7JHP8, Q9GJT5, P51905, Q6PWI2, O35899, Q9XT49, Q9GMA5, P31645, Q9MYX0, P31652, Q60857, Q5NCR6	**Q61IM9**, **Q7QJ94**, **Q96KH8**, **Q6QU62**, **Q7PSC9**
Renal system (24)	O88575, Q9NP91, Q64093, Q8VDB9, Q96N87, O88576, Q62687	Q9GZN6, Q8IYV4, Q9H1V8, Q8BJI1, P31662, Q8BG16, Q08469, Q80XW8, Q9XS59, Q5R9C2, Q9H2J7, Q68CX0, **Q5XIV7**, Q66HV0, Q6PBA3, Q66J35, Q66J60
Amino acid 1 (18)	Q7YZB6, Q28039, Q5TAB8, P48067, Q5TAB9, Q5TAC0, P28571, P28572, Q99884, Q6PGE7, P28573, Q9Y345, Q761V0, P58295, Q9JMA9, Q9UN76, Q5H942	**Q5H942**
Amino acid 2 (5)	O76188, Q9U5A9	Q7KR41, Q9V690, Q9W4C5
Prokaryotic (44)	2A65 (LeuT$_{Aa}$)	TP0023, CPn0290, CT231, FN1944, FN1989, aq_2077, PAB1850, H1372, Cj0601c, PM0380, HI1690, 45320, Cj0934c, Cj0935c, HP0498, jhp0450, HP0497, jhp0449, MJ1319, Q58715, MA1518, AF1995, VC2012, BH3439, Cgl1001, NMA0470, NMB1975, PM0718, HI0736, P44849, MA0901, VC1669, VC2283, VNG1526G, VNG1528G, BH1128, SA0417, NMA1961, NMB1707, lin1873, BS_yhdH, BH0217, BS_yocR
