# Supplementary material for: Assessing the ability of sequence-based methods to provide functional insight within membrane integral proteins: a case study analyzing the neurotransmitter/Na+ symporter family
Source: BMC Bioinformatics. 2007 Oct 17;8:397. doi: 10.1186/1471-2105-8-397 (PMC2194793; doi:10.1186/1471-2105-8-397)

Prediction method performance. Accuracy is plotted against coverage for each method considered. A perfect method would have accuracy = coverage = 1.00. Overall performance is simply calculated from the Cartesian distance from each point on the graph and the perfect value (normalized from zero to unity). The distances are then subtracted from one such that larger values indicate better methods. The Union method (PM + FPE) has the best coverage, whereas the Intersect5 method has the best accuracy. The best overall method is Intersect3, which has coverage of 0.56 and accuracy of 0.44. SDPpred, which has the worst overall performance, is not used in the calculation of the Intersect results.

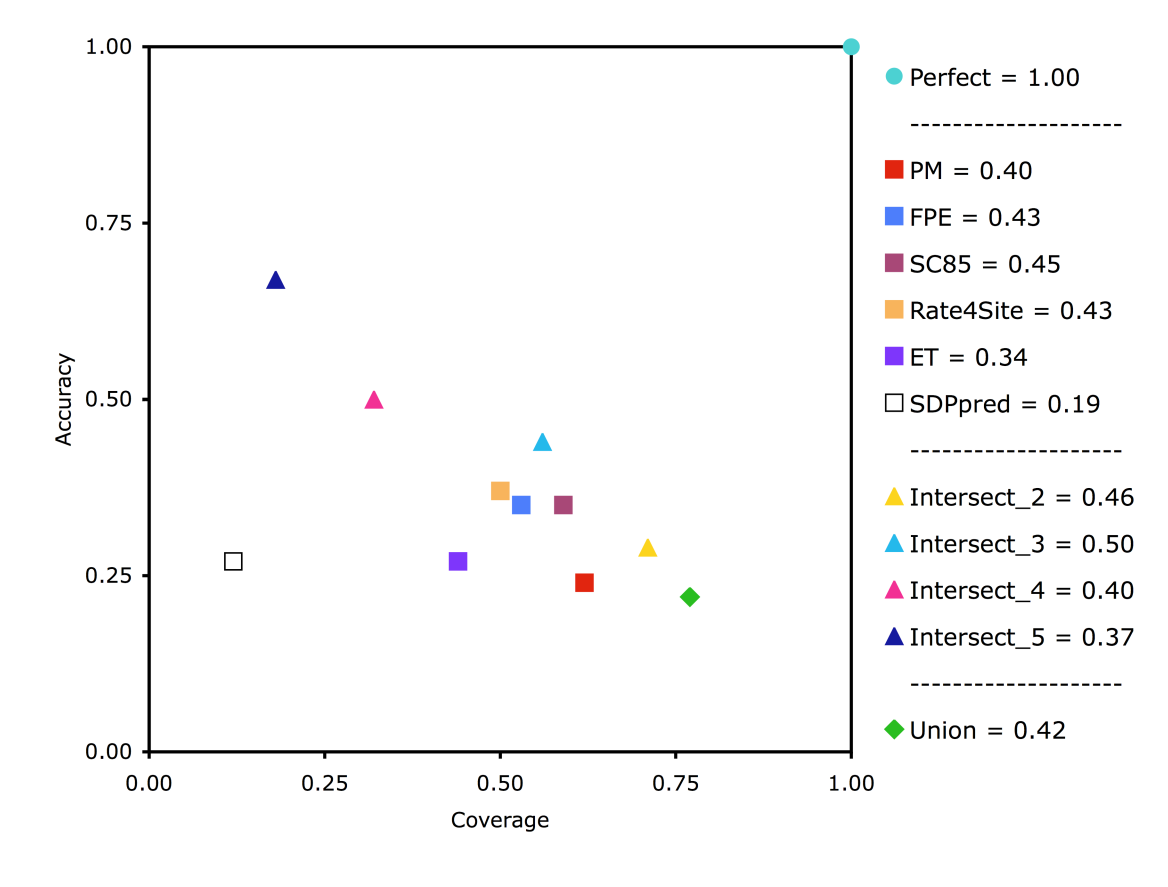

Supplement: Additional file 3 — Prediction method performance figure. This file contains a plot of accuracy vs. coverage for each prediction method considered. Overall performance values are also provided. [file 1471-2105-8-397-S3.pdf]
