# Supplementary material for: Assessing the ability of sequence-based methods to provide functional insight within membrane integral proteins: a case study analyzing the neurotransmitter/Na+ symporter family
Source: BMC Bioinformatics. 2007 Oct 17;8:397. doi: 10.1186/1471-2105-8-397 (PMC2194793; doi:10.1186/1471-2105-8-397)
Supplement: Additional file 4 — Prediction summary figure. This file annotates the LeuTAa sequence with the predictions of each unique method. The sequence is color-coded based on the Intersect results. [file 1471-2105-8-397-S4.pdf]

Prediction summary of each unique method. An asterisk above the corresponding alignment position indicates each functional site (red = leucine/Na^+ binding site, green = predicted cytoplasmic gate residue, and purple = predicted extracellular/periplasmic gate residue). Shading of the $LeuT_{Aa}$ sequence indicates sites that are predicted by one (cyan), two (green), three (yellow) four (red), or five (magenta) prediction techniques. As discussed in the text, SDPpred is not considered in the intersect results due to poor overall performance, nor is it considered in the color-coding here. The technique that predicts each site is noted below each alignment position (P = Phylogenetic Motif, F = False Positive Expectation, E = Evolutionary Trace, S = Site Conservation, R = Rate4Site, and D = SDPpred). Transmembrane and other helices are indicated as well. Numbering is based on the $LeuT_{Aa}$ structure.

```
         10        20        30        40        50        60        70
....*..*.|....:....********..*....:....|....:.*..|....:....|.*..:....|
xxxxREHWATRLGLILAMAGNAVGLGNFLRFPVQAAENGGGAFMIPYIIAFLLVGIPLMWIEWAMGRYGG
         |--- TM1a --|  |--- TM1b --|   |------------ TM2 -----------|
            PPPPPPPP    PPPPPPPPPPPPPPPPPPPPPPP         PPPPPPPPPP
                     FFFFFFFFFFFF  FFFFFFFFFFFF              FFFFF
             E   E E   EE E EE EE       E E  EE       E E E  E    E
    S  S           S    SSS SSSSS    SSSSSS   S       S S S  S
                   R R RRRR RRRR      RR RR   R       R      R   R
             D    D     D

         80        90        100       110       120       130       140
....:....|....:....|....:....|...*:..*.|....:....|....:....|....:....|
AQGHGTTPAIFYLLWRNRFAKILGVFGLWIPLVVAIYYVYIESWTLGFAIKFLVGLVPEPPPxxTDPDSI
     |- IL1 -|   |--------------- TM3 ---------------|            |---
                                          PPPPP
                                    FFFFF
    E                                      E
           S     S     S             S     S   S
                       R             R     R
  D                                   D

         150       160       170       180       190       200       210
....:....|....:....|....:....|....:....|....:....|....:....|....:....|
LRPFKEFLYSYIGVPKGDEPILKPSLFAYIVFLITMFINVSILIRGISKGIERFAKIAMPTLFILAVFLV
-- EL2 ----|             |------ TM4 -----|       |-------- TM5 ------
    PPPPPPP                               PPPPPP P PPPP
    FFFFF
                                                 EE
                     S                       S      S
                                             S    RRR     R
   D D D        D DDD D   D              D       D D      DD
```

```
         220       230       240       250       260       270       280
....:....|....:....|....:....|....:....*.***:*****....:.**.|....:....|
IRVFLLETPNGTAADGLNFLWTPDFEKLKDPGVWIAAVGQIFFTLSLGFGAIITYASYVRKDQDIVLSGL
--|         |- EL3 -|         |---- TM6a ---|     |TM6b|         |----
    P    PPPP
                                       PPPPPPPPPPPPPP
                                       FFFFFFF
                                EE  E  E   E     E E E  EEE E
         S S   S                SS  S  S SS      S      SS
         R R   R                    R  R R              R R
                                            DD    D   DD    D

         290       300       310       320       330       340       350
....:*...*....:....|....:....|....:....|....:....|....:....|....:....|
TAATLNEKAEVILGGSISIPAAVAFFGVANAVAIAKAGAFNLGFITLPAIFSQTAGGTFLGFLWFFLLFF
-------- TM7 ------------| |-- EL4a --| |-- EL4b -|     |-------------
 PPPPP PPPPP    PPPPP                             PPPPP
                                                               FFFFF
              EE    E                   E  E  E                EE E
     S             S   S              S  S S                   SS   S
     R  R     R    R                  R    R                   RR
                            D       D         D   D D

         360       370       380       390       400       410       420
*..**...*|....:...*|....:....|....:....|....:....|...*:....|....:....|
AGLTSSIAIMQPMIAFLEDELKLSRKHAVLWTAAIVFFSAHLVMFLNKSLDEMDFWAGTIGVVFFGLTEL
-- TM8 ----------|      |------- TM9 -------|   |--------- TM10 ------
  PPPPP                                P  PPPPPP PPPPPPPPPPP
FFFFF
E  E   E          E                     EE  E
 S  S     S       S                                  S
R  RR                   R
D  D   DD               D

         430       440       450       460       470       480       490
....:....|....:....|....:....|....:....|....:....|....:....|....:....|
IIFFWIFGADKAWEEINRGGIIKVPRIYYYVMRYITPAFLAVLLVVWAREYIPKIMEETHWTVWITRFYI
---|    |- IL5 -|         |------------ TM11 -----------|     |-------



                                    S

                 D   D                 D

         500       510
....:....|....:....|....:
IGLFLFLTFLVFLAERRRNHESAGT
----- TM12 -----------|
```
