# Supplementary material for: Assessing the ability of sequence-based methods to provide functional insight within membrane integral proteins: a case study analyzing the neurotransmitter/Na+ symporter family
Source: BMC Bioinformatics. 2007 Oct 17;8:397. doi: 10.1186/1471-2105-8-397 (PMC2194793; doi:10.1186/1471-2105-8-397)

Binding pocket residues. Shown are the leucine substrate (center), two sodium ions (blue), and the three residues not predicted by any of the bioinformatic methods. Asn21 makes van der Waals contacts with the leucine substrate via backbone atoms, whereas the hydrophobic sidechains of Val104 and Ile359 form the base of the hydrophobic pocket in which the leucine sits. While positions 104 and 359 are not strictly conserved, the positions are generally hydrophobic. As such, it is natural to assume that amphipathic substrates (other amino acids and the biogenic amines) reside in the pocket in a similar fashion. However, the osmolyte substrates are charged on both ends, so it remains unclear how this is reconciled with the largely hydrophobic binding pocket.

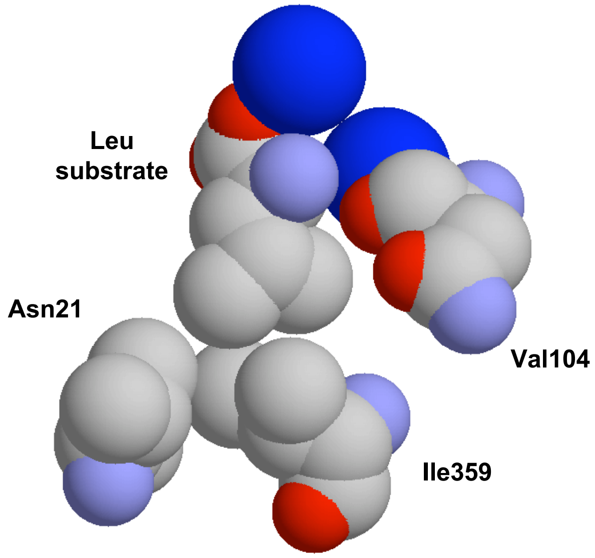

Supplement: Additional file 5 — Leucine-binding pocket figure. This file contains a figure showing the leucine substrate and the three binding pocked residues that are not predicted by any method used herein. [file 1471-2105-8-397-S5.pdf]
