# Supplementary material for: Assessing the ability of sequence-based methods to provide functional insight within membrane integral proteins: a case study analyzing the neurotransmitter/Na+ symporter family
Source: BMC Bioinformatics. 2007 Oct 17;8:397. doi: 10.1186/1471-2105-8-397 (PMC2194793; doi:10.1186/1471-2105-8-397)
Supplement: Additional file 7 — Alignment supplementary material. This file contains the sum-of-pairs scores for each alignment pair and a series of tables that detail the predictions of each method as a function of alignment input. [file 1471-2105-8-397-S7.pdf]

Sum-of-pairs scores comparing each alignment pair. The matrix is not exactly symmetrical because the raw scores are divided by the number of aligned pairs within the reference alignment (listed on the vertical).

	MUSCLE	MAFFT	Probalign	Probcons
**MUSCLE**	1.00	0.86	0.86	0.86
**MAFFT**	0.87	1.00	0.94	0.91
**Probalign**	0.87	0.94	1.00	0.91
**Probcons**	0.86	0.91	0.91	1.00

Sensitivity of PM predictions to alignment input. This table compares the PM functional site predictions across the four different alignments.

Site	Description	MUSCLE	MAFFT	Probalign	Probcons
Arg5	Cytoplasmic gate				
Trp8	Cytoplasmic gate				
Gly20	Na^+ binding site	Yes	Yes	Yes	Yes
Asn21	Leu binding site		Yes	Yes	Yes
Ala22	Leu & Na^+ binding sites		Yes	Yes	Yes
Val23	Na^+ binding site & unwound TM1		Yes	Yes	Yes
Gly24	Leu binding site & unwound TM1		Yes	Yes	Yes
Leu25	Leu binding site	Yes	Yes	Yes	Yes
Gly26	Leu binding site	Yes	Yes	Yes	Yes
Asn27	Na^+ binding site	Yes	Yes	Yes	Yes
Arg30	Extracellular gate	Yes	Yes	Yes	Yes
Tyr47	Extracellular gate	Yes	Yes	Yes	Yes
Glu62	Stabilizes unwound region in TM6	Yes			
Val104	Leu binding site				
Tyr108	Leu binding site				
Gln250	Extracellular gate	Yes	Yes	Yes	Yes
Phe252	Leu binding site	Yes	Yes	Yes	Yes
Phe253	Leu binding site	Yes	Yes	Yes	Yes
Thr254	Leu & Na^+ binding sites	Yes			
Ser256	Leu binding site & unwound TM6	Yes			
Leu257	Unwound TM6	Yes			
Gly258	Unwound TM6	Yes			
Phe259	Leu binding site & unwound TM6	Yes			
Gly260	Unwound TM6	Yes			
Ser267	Cytoplasmic gate		Yes	Yes	Yes
Tyr268	Cytoplasmic gate		Yes	Yes	Yes
Asn286	Na^+ binding site	Yes			
Glu290	Extracellular gate	Yes			
Ala351	Na^+ binding site				
Thr354	Na^+ binding site	Yes			
Ser355	Leu & Na^+ binding sites	Yes			
Ile359	Leu binding site		Yes	Yes	Yes
Asp369	Cytoplasmic gate				
Asp404	Extracellular gate	Yes			
**Number correct**		**21**	**16**	**16**	**16**
**Total number of predictions**		**126**	**112**	**94**	**104**
**Absolute accuracy**		**0.17**	**0.14**	**0.17**	**0.15**

Sensitivity of FPE predictions to alignment input. This table compares the FPE functional site predictions across the four different alignments.

Site	Description	MUSCLE	MAFFT	Probalign	Probcons
Arg5	Cytoplasmic gate				
Trp8	Cytoplasmic gate				
Gly20	Na^+ binding site				
Asn21	Leu binding site				
Ala22	Leu & Na^+ binding sites	Yes	Yes	Yes	Yes
Val23	Na^+ binding site & unwound TM1	Yes	Yes	Yes	Yes
Gly24	Leu binding site & unwound TM1	Yes	Yes	Yes	Yes
Leu25	Leu binding site	Yes	Yes	Yes	Yes
Gly26	Leu binding site	Yes	Yes	Yes	Yes
Asn27	Na^+ binding site	Yes	Yes	Yes	Yes
Arg30	Extracellular gate	Yes	Yes	Yes	Yes
Tyr47	Extracellular gate	Yes	Yes	Yes	Yes
Glu62	Stabilizes unwound region in TM6	Yes		Yes	Yes
Val104	Leu binding site				
Tyr108	Leu binding site	Yes	Yes	Yes	Yes
Gln250	Extracellular gate	Yes	Yes	Yes	Yes
Phe252	Leu binding site	Yes	Yes	Yes	Yes
Phe253	Leu binding site	Yes	Yes	Yes	Yes
Thr254	Leu & Na^+ binding sites	Yes	Yes	Yes	Yes
Ser256	Leu binding site & unwound TM6	Yes	Yes	Yes	Yes
Leu257	Unwound TM6				
Gly258	Unwound TM6				
Phe259	Leu binding site & unwound TM6				
Gly260	Unwound TM6				
Ser267	Cytoplasmic gate				
Tyr268	Cytoplasmic gate				
Asn286	Na^+ binding site				
Glu290	Extracellular gate				
Ala351	Na^+ binding site	Yes	Yes	Yes	Yes
Thr354	Na^+ binding site	Yes	Yes	Yes	Yes
Ser355	Leu & Na^+ binding sites	Yes	Yes	Yes	Yes
Ile359	Leu binding site				
Asp369	Cytoplasmic gate				
Asp404	Extracellular gate				
**Number correct**		**18**	**17**	**18**	**18**
**Total number of predictions**		**51**	**51**	**51**	**51**
**Absolute accuracy**		**0.35**	**0.33**	**0.35**	**0.35**

Sensitivity of SC85 predictions to alignment input. This table compares the SC85 functional site predictions across the four different alignments.

Site	Description	MUSCLE	MAFFT	Probalign	Probcons
Arg5	Cytoplasmic gate	Yes	Yes	Yes	Yes
Trp8	Cytoplasmic gate	Yes	Yes	Yes	Yes
Gly20	Na^+ binding site	Yes	Yes	Yes	Yes
Asn21	Leu binding site				
Ala22	Leu & Na^+ binding sites				
Val23	Na^+ binding site & unwound TM1				
Gly24	Leu binding site & unwound TM1				
Leu25	Leu binding site	Yes	Yes	Yes	Yes
Gly26	Leu binding site	Yes			
Asn27	Na^+ binding site	Yes	Yes	Yes	Yes
Arg30	Extracellular gate	Yes	Yes	Yes	Yes
Tyr47	Extracellular gate	Yes	Yes	Yes	Yes
Glu62	Stabilizes unwound region in TM6	Yes	Yes	Yes	Yes
Val104	Leu binding site				
Tyr108	Leu binding site	Yes	Yes	Yes	Yes
Gln250	Extracellular gate	Yes	Yes	Yes	Yes
Phe252	Leu binding site	Yes	Yes	Yes	Yes
Phe253	Leu binding site	Yes	Yes	Yes	Yes
Thr254	Leu & Na^+ binding sites				
Ser256	Leu binding site & unwound TM6				
Leu257	Unwound TM6				
Gly258	Unwound TM6				
Phe259	Leu binding site & unwound TM6				
Gly260	Unwound TM6	Yes	Yes	Yes	Yes
Ser267	Cytoplasmic gate	Yes	Yes	Yes	Yes
Tyr268	Cytoplasmic gate	Yes	Yes	Yes	Yes
Asn286	Na^+ binding site	Yes	Yes	Yes	Yes
Glu290	Extracellular gate				
Ala351	Na^+ binding site				
Thr354	Na^+ binding site				
Ser355	Leu & Na^+ binding sites	Yes	Yes	Yes	Yes
Ile359	Leu binding site				
Asp369	Cytoplasmic gate	Yes	Yes	Yes	Yes
Asp404	Extracellular gate	Yes	Yes	Yes	Yes
**Number correct**		**20**	**19**	**19**	**19**
**Total number of predictions**		**58**	**53**	**55**	**55**
**Accuracy**		**0.35**	**0.36**	**0.35**	**0.35**

Sensitivity of Rate4Site predictions to alignment input. This table compares the Rate4Site (conservation level = 9) functional site predictions across the four different alignments.

Site	Description	MUSCLE	MAFFT	Probalign	Probcons
Arg5	Cytoplasmic gate				
Trp8	Cytoplasmic gate				
Gly20	Na^+ binding site	Yes	Yes	Yes	Yes
Asn21	Leu binding site				
Ala22	Leu & Na^+ binding sites	Yes	Yes	Yes	Yes
Val23	Na^+ binding site & unwound TM1				
Gly24	Leu binding site & unwound TM1	Yes	Yes	Yes	Yes
Leu25	Leu binding site	Yes	Yes	Yes	Yes
Gly26	Leu binding site	Yes	Yes	Yes	Yes
Asn27	Na^+ binding site	Yes	Yes	Yes	Yes
Arg30	Extracellular gate	Yes	Yes	Yes	Yes
Tyr47	Extracellular gate	Yes	Yes	Yes	Yes
Glu62	Stabilizes unwound region in TM6	Yes	Yes	Yes	Yes
Val104	Leu binding site				
Tyr108	Leu binding site	Yes	Yes	Yes	Yes
Gln250	Extracellular gate	Yes	Yes	Yes	Yes
Phe252	Leu binding site	Yes	Yes	Yes	Yes
Phe253	Leu binding site				
Thr254	Leu & Na^+ binding sites				
Ser256	Leu binding site & unwound TM6				
Leu257	Unwound TM6				
Gly258	Unwound TM6				
Phe259	Leu binding site & unwound TM6				
Gly260	Unwound TM6				
Ser267	Cytoplasmic gate	Yes	Yes	Yes	Yes
Tyr268	Cytoplasmic gate				
Asn286	Na^+ binding site	Yes	Yes	Yes	Yes
Glu290	Extracellular gate				
Ala351	Na^+ binding site	Yes	Yes	Yes	Yes
Thr354	Na^+ binding site	Yes	Yes	Yes	Yes
Ser355	Leu & Na^+ binding sites	Yes	Yes	Yes	Yes
Ile359	Leu binding site				
Asp369	Cytoplasmic gate				
Asp404	Extracellular gate				
**Number correct**		**17**	**17**	**17**	**17**
**Total number of predictions**		**46**	**47**	**43**	**41**
**Accuracy**		**0.37**	**0.36**	**0.40**	**0.42**

Sensitivity of ET predictions to alignment input. This table compares the ET (cut level = 12) functional site predictions across the four different alignments.

Site	Description	MUSCLE	MAFFT	Probalign	Probcons
Arg5	Cytoplasmic gate				
Trp8	Cytoplasmic gate				
Gly20	Na^+ binding site	Yes	Yes	Yes	Yes
Asn21	Leu binding site				
Ala22	Leu & Na^+ binding sites				
Val23	Na^+ binding site & unwound TM1				
Gly24	Leu binding site & unwound TM1	Yes	Yes	Yes	Yes
Leu25	Leu binding site	Yes	Yes	Yes	Yes
Gly26	Leu binding site				
Asn27	Na^+ binding site	Yes	Yes	Yes	Yes
Arg30	Extracellular gate	Yes	Yes	Yes	Yes
Tyr47	Extracellular gate	Yes	Yes	Yes	Yes
Glu62	Stabilizes unwound region in TM6	Yes	Yes	Yes	Yes
Val104	Leu binding site				
Tyr108	Leu binding site				
Gln250	Extracellular gate	Yes	Yes	Yes	Yes
Phe252	Leu binding site				
Phe253	Leu binding site				
Thr254	Leu & Na^+ binding sites	Yes	Yes	Yes	Yes
Ser256	Leu binding site & unwound TM6				
Leu257	Unwound TM6				
Gly258	Unwound TM6				
Phe259	Leu binding site & unwound TM6				
Gly260	Unwound TM6	Yes	Yes	Yes	Yes
Ser267	Cytoplasmic gate	Yes	Yes	Yes	Yes
Tyr268	Cytoplasmic gate	Yes	Yes	Yes	Yes
Asn286	Na^+ binding site				
Glu290	Extracellular gate				
Ala351	Na^+ binding site	Yes	Yes	Yes	Yes
Thr354	Na^+ binding site	Yes	Yes	Yes	Yes
Ser355	Leu & Na^+ binding sites				
Ile359	Leu binding site				
Asp369	Cytoplasmic gate	Yes	Yes	Yes	
Asp404	Extracellular gate				
**Number correct**		**15**	**15**	**15**	**14**
**Total number of predictions**		**56**	**50**	**62**	**55**
**Accuracy**		**0.27**	**0.30**	**0.24**	**0.26**

Sensitivity of SDP predictions to alignment input. This table compares the SDPpred (z-score threshold = 6) functional site predictions across the four different alignments.

Site	Description	MUSCLE	MAFFT	Probalign	Probcons
Arg5	Cytoplasmic gate				
Trp8	Cytoplasmic gate				
Gly20	a$^+$ binding site				
Asn21	Leu binding site				
Ala22	Leu & Na$^+$ binding sites				
Val23	Na$^+$ binding site & unwound TM1				
Gly24	Leu binding site & unwound TM1	Yes	Yes	Yes	Yes
Leu25	Leu binding site				
Gly26	Leu binding site				
Asn27	Na$^+$ binding site				
Arg30	Extracellular gate				
Tyr47	Extracellular gate				
Glu62	Stabilizes unwound region in TM6				
Val104	Leu binding site				
Tyr108	Leu binding site				
Gln250	Extracellular gate				
Phe252	Leu binding site				
Phe253	Leu binding site				
Thr254	Leu & Na$^+$ binding sites				
Ser256	Leu binding site & unwound TM6	Yes	Yes	Yes	Yes
Leu257	Unwound TM6				
Gly258	Unwound TM6				
Phe259	Leu binding site & unwound TM6				
Gly260	Unwound TM6				
Ser267	Cytoplasmic gate				
Tyr268	Cytoplasmic gate				
Asn286	Na$^+$ binding site				
Glu290	Extracellular gate				
Ala351	Na$^+$ binding site	Yes	Yes	Yes	Yes
Thr354	Na$^+$ binding site	Yes	Yes	Yes	Yes
Ser355	Leu & Na$^+$ binding sites				
Ile359	Leu binding site				
Asp369	Cytoplasmic gate				
Asp404	Extracellular gate				
**Number correct**		**4**	**4**	**4**	**4**
**Total number of predictions**		**15**	**17**	**18**	**21**
**Accuracy**		**0.26**	**0.24**	**0.22**	**0.19**
