# Supplementary material for: Assessing the ability of sequence-based methods to provide functional insight within membrane integral proteins: a case study analyzing the neurotransmitter/Na+ symporter family
Source: BMC Bioinformatics. 2007 Oct 17;8:397. doi: 10.1186/1471-2105-8-397 (PMC2194793; doi:10.1186/1471-2105-8-397)

Phylogenetic motif prediction summary. Phylogenetic motifs (PMs) are identified from the phylogenetic similarity spectrum. Any window scoring past the phylogenetic similarity z-score (PSZ) threshold is part of a PM. All overlapping windows are grouped into a single PM. In this case, the PSZ threshold is set to -1.4, which is sufficient to separate signal from noise. In this example, eleven PMs are identified.

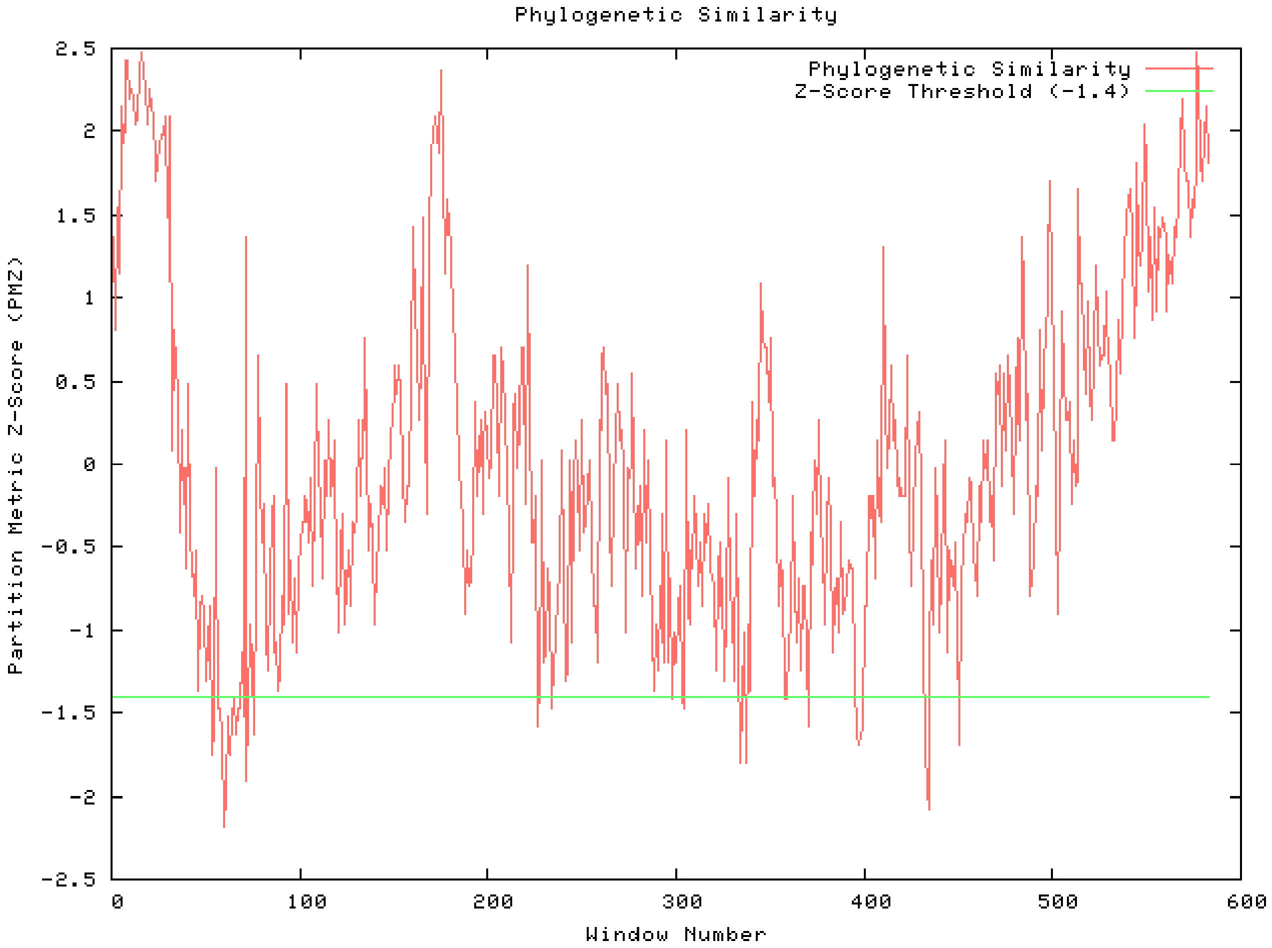

FPE prediction summary. Traditional motifs (i.e., regions with reduced sequence variability) are identified in a similar manner as the phylogenetic motifs. For each window, a false positive expectation (FPE) is calculated. All ln(FPE z-scores) below some threshold (here -2.2) are put forth. As with the phylogenetic motifs, all overlapping windows scoring past the threshold are grouped into a single motif. In this example, seven FPE motifs are identified.

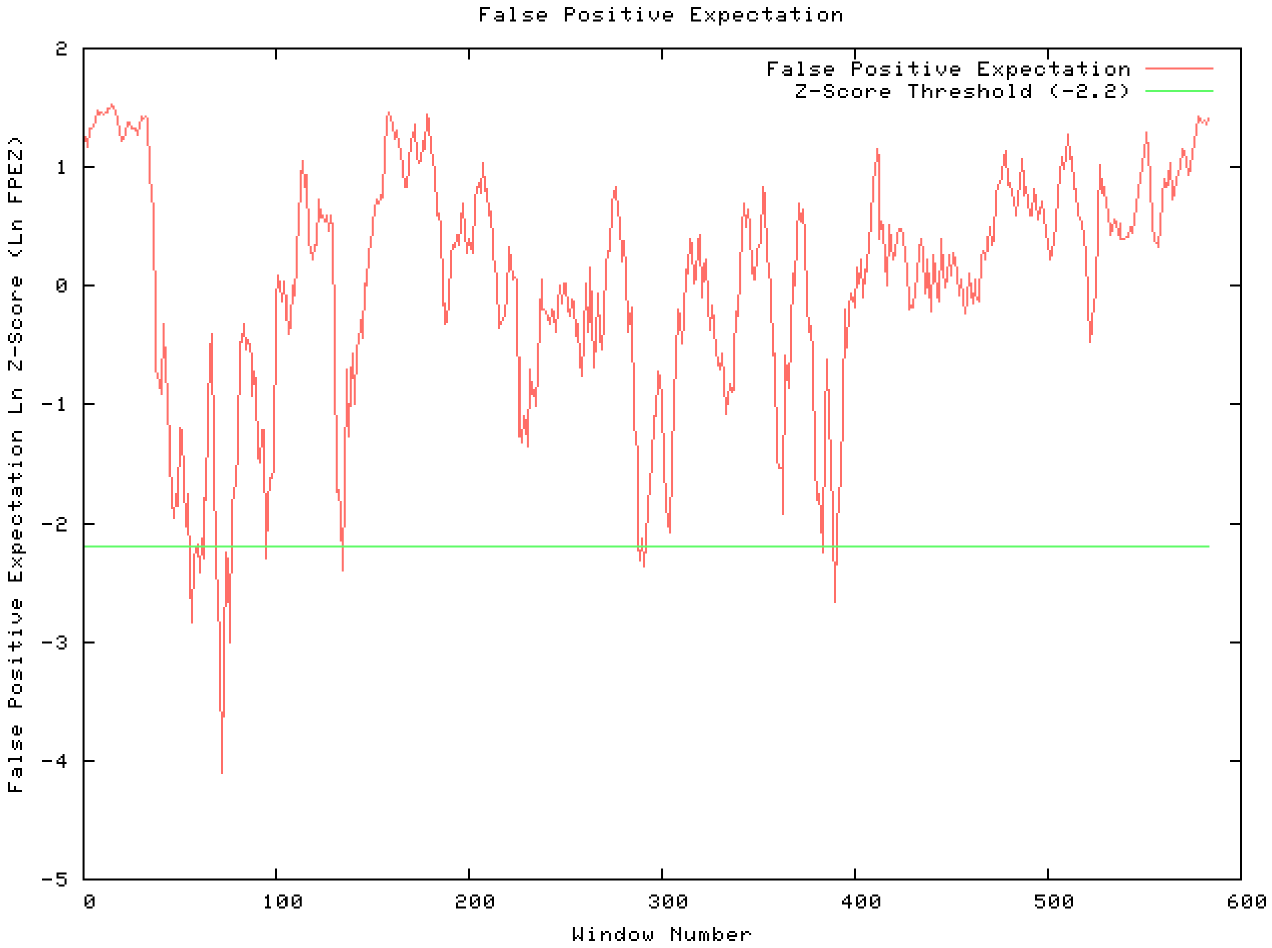

PSZ vs. FPE. Phylogenetic similarity z-scores vs. false positive expectation for each window within the MUSCLE alignment. As can be seen, there is a clear positive correlation between the two metrics; however, there are several instances when the methods give varying results, which is exactly consistent with the results presented in Table 4.

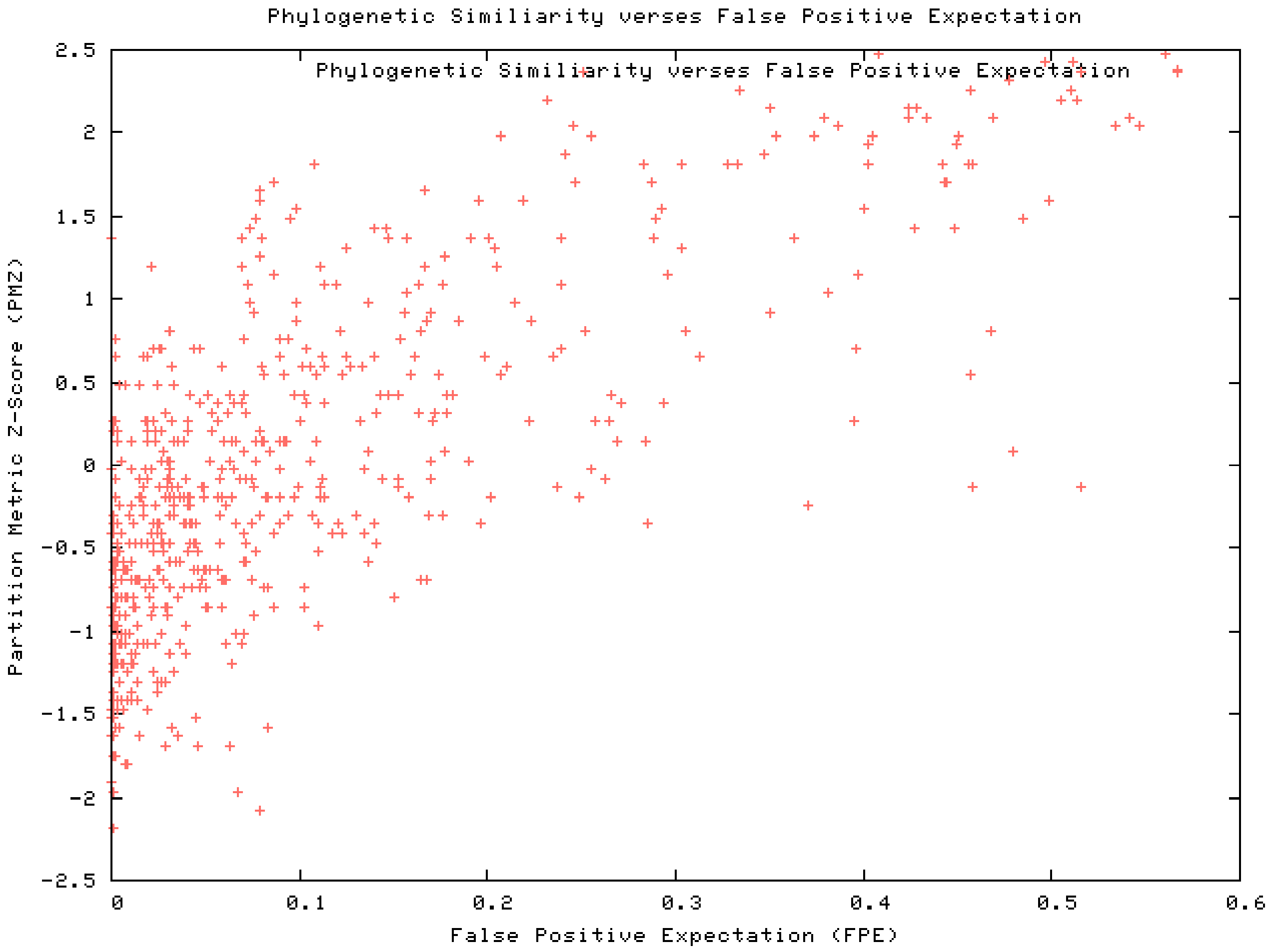

Supplement: Additional file 8 — MINER supplemental figures. This file contains supplementary figures associated with MINER. [file 1471-2105-8-397-S8.pdf]
