# Supplementary material for: Assessing the ability of sequence-based methods to provide functional insight within membrane integral proteins: a case study analyzing the neurotransmitter/Na+ symporter family
Source: BMC Bioinformatics. 2007 Oct 17;8:397. doi: 10.1186/1471-2105-8-397 (PMC2194793; doi:10.1186/1471-2105-8-397)
Supplement: Additional file 9 — Prediction threshold summary. This file contains descriptions of performance as a function of threshold level. [file 1471-2105-8-397-S9.pdf]

Site conservation threshold results. Coverage and accuracy of the site conservation approach using the MUSCLE alignment at various conservation thresholds are provided. The best overall performing threshold (highlighted in red) is solely discussed within the text.

Method	Coverage	Absolute Accuracy	Overall Performance
100%	0.00 (0)	0.00 (4)	0.00
95%	0.15 (5)	0.24 (19)	0.19
90%	0.29 (10)	0.30 (34)	0.29
**85%**	**0.59 (20)**	**0.35 (58)**	**0.46**
80%	0.59 (20)	0.28 (71)	0.41
75%	0.59 (20)	0.23 (88)	0.38

Rate4Site threshold results. Coverage and accuracy of Rate4Site using the MUSCLE alignment at various conservation levels is provided. While the overall performance at threshold level 8 is the best, the text solely discusses the results at level 9 (highlighted in red) since the accuracy of level 8 is greatly diminished.

Method	Coverage	Absolute Accuracy	Overall Performance
**Level 9**	**0.50 (17)**	**0.37 (46)**	**0.43**
Level 8	0.82 (28)	0.25 (114)	0.45
Level 7	0.88 (30)	0.16 (192)	0.40
Level 6	0.97 (33)	0.13 (262)	0.38

Evolutionary trace threshold results. Coverage and accuracy of the ET approach at various tree cut levels using the MUSCLE alignment are provided. While the overall performance at cut level 14 is marginally the best, the text solely discusses the results at cut level 12 (highlighted in red) since its accuracy is substantially better.

Method	Coverage	Absolute Accuracy	Overall Performance
ET (cut level = 8)	0.15 (5)	0.24 (21)	0.19
ET (cut level = 10)	0.32 (11)	0.29 (38)	0.30
**ET (cut level = 12)**	**0.44 (15)**	**0.27 (56)**	**0.35**
ET (cut level = 14)	0.56 (19)	0.21 (89)	0.36
ET (cut level = 16)	0.59 (20)	0.17 (116)	0.35

SDPpred threshold results. Coverage and accuracy of SDPpred using the MUSCLE alignment at various conservation levels are provided. The best overall performing threshold (highlighted in red) is solely discussed within the text.

Method	Coverage	Absolute Accuracy	Overall Performance
**Z-score 6**	**0.12 (4)**	**0.27 (15)**	**0.19**
Z-score 5	0.12 (5)	0.20 (25)	0.17
Z-score 4	0.15 (5)	0.13 (38)	0.14
